# Characterization of Flow Dynamics in the Pulmonary Bifurcation of Patients With Repaired Tetralogy of Fallot: A Computational Approach

**DOI:** 10.3389/fcvm.2021.703717

**Published:** 2021-09-30

**Authors:** Maria Boumpouli, Emilie L. Sauvage, Claudio Capelli, Silvia Schievano, Asimina Kazakidi

**Affiliations:** ^1^Department of Biomedical Engineering, University of Strathclyde, Glasgow, United Kingdom; ^2^Institute of Cardiovascular Science and Great Ormond Street Hospital for Children, NHS Foundation Trust, University College London, London, United Kingdom

**Keywords:** hemodynamics, computational fluid dynamics, congenital heart disease, tetralogy of Fallot, pulmonary bifurcation

## Abstract

The hemodynamic environment of the pulmonary bifurcation is of great importance for adult patients with repaired tetralogy of Fallot (rTOF) due to possible complications in the pulmonary valve and narrowing of the left pulmonary artery (LPA). The aim of this study was to computationally investigate the effect of geometrical variability and flow split on blood flow characteristics in the pulmonary trunk of patient-specific models. Data from a cohort of seven patients was used retrospectively and the pulmonary hemodynamics was investigated using averaged and MRI-derived patient-specific boundary conditions on the individualized models, as well as a statistical mean geometry. Geometrical analysis showed that curvature and tortuosity are higher in the LPA branch, compared to the right pulmonary artery (RPA), resulting in complex flow patterns in the LPA. The computational analysis also demonstrated high time-averaged wall shear stress (TAWSS) at the outer wall of the LPA and the wall of the RPA proximal to the junction. Similar TAWSS patterns were observed for averaged boundary conditions, except for a significantly modified flow split assigned at the outlets. Overall, this study enhances our understanding about the flow development in the pulmonary bifurcation of rTOF patients and associates some morphological characteristics with hemodynamic parameters, highlighting the importance of patient-specificity in the models. To confirm these findings, further studies are required with a bigger cohort of patients.

## Introduction

Tetralogy of Fallot is one of the most common cyanotic congenital heart diseases with an estimated prevalence of 1 in 3,000 live births (1). This condition is characterized by four primary defects: a ventricular septal defect, an overriding aorta, pulmonary stenosis, and a right ventricular hypertrophy (2). Surgical intervention is required in these patients and the non-preservative traditional transannular patch repair (TAP) is the predominant approach used to repair pulmonary insufficiency (3). However, long term complications, including right ventricular dilatation and dysfunction, pulmonary regurgitation (PR), residual right ventricular outflow tract (RVOT) obstruction, kinking of the left pulmonary artery (LPA) and atrial tachyarrhythmia (4), are common in the repaired population. Pulmonary valve replacement is performed in the repaired Tetralogy of Fallot (rTOF) population to prevent ongoing volume overloading and dilation of the right ventricle, and is recommended prior to the significant clinical symptoms to increase the likelihood of successfully RV remodeling; nevertheless, there is no common consensus on the reliability of current indices to determine the right timing for intervention (4–6). Understanding the hemodynamic environment in the pulmonary bifurcation of rTOF patients is therefore crucial to foresee long term outcomes of the interventions.

The advancement of medical imaging techniques over the past decades has allowed the application of computational fluid dynamics for the assessment of blood flow in arteries. Magnetic resonance (MR) and computed tomography (CT) has enabled the reconstruction of patient-specific models of detailed anatomic variations (7, 8), while 2D phase-contrast MRI is used to extract flow information from a vessel's cross-sectional lumen (9, 10). Such techniques are now routinely performed and are used to assess the hemodynamic conditions in both healthy (11) and diseased subjects (12), under normal or exercise conditions (13), in order to compare morphological and geometrical features (14) to predict surgical outcomes (15–17) and facilitate the treatment and device design (18).

Patients with tetralogy of Fallot have a significant variability in the anatomy of the pulmonary arteries and it is therefore crucial to characterize the effect of geometric parameters in the hemodynamic environment in these patients. Although the pulmonary bifurcation in healthy subjects is found hemodynamically efficient (11), studies in TOF models have indicated reversal of flow in the left pulmonary artery (LPA) and an influence of the branching angles in pulmonary regurgitation (19–21). Recently, Louvelle et al. (22) tried to link geometric parameters such as diameter, length, tortuosity and the angle of the branches, in TOF patients repaired with preservative and non-preservative techniques with the hemodynamic characteristics that can influence reversal of flow.

In this computational study, the blood flow environment in patient-specific models of rTOF patients is described. This study is a continuation of a previous work performed in idealized models in relation to TOF patients, where the impact of morphology and the role of the stagnation point in the wall shear stress distribution were discussed (23). The authors identified a correlation between the wall shear stress distribution, the pressure difference in the daughter branches and the flow splits. To further explore the above findings, the patient-specific models are analyzed and parameters such as the angle of branches, curvature, tortuosity, and planarity are reported. The hemodynamic environment of the geometries is also examined with the aim to better understand the flow development in the pulmonary junction of these patients and potentially correlate specific geometrical features with blood flow patterns. The layout of this work is as follows: First the methodology is presented (section Methodology), followed by the results (section Results). In discussion we summarize the main findings of this work and we make comparisons with previous studies (section Discussion), and we end with a brief conclusion.

## Methodology

In this section, we introduce the patients cohort, the extraction of flow data, and the segmentation of the models. Additionally, we present the computational model setup, and we explain how the parameters for the geometry and flow characterization were computed.

### Patients Population

**Figure 1:**
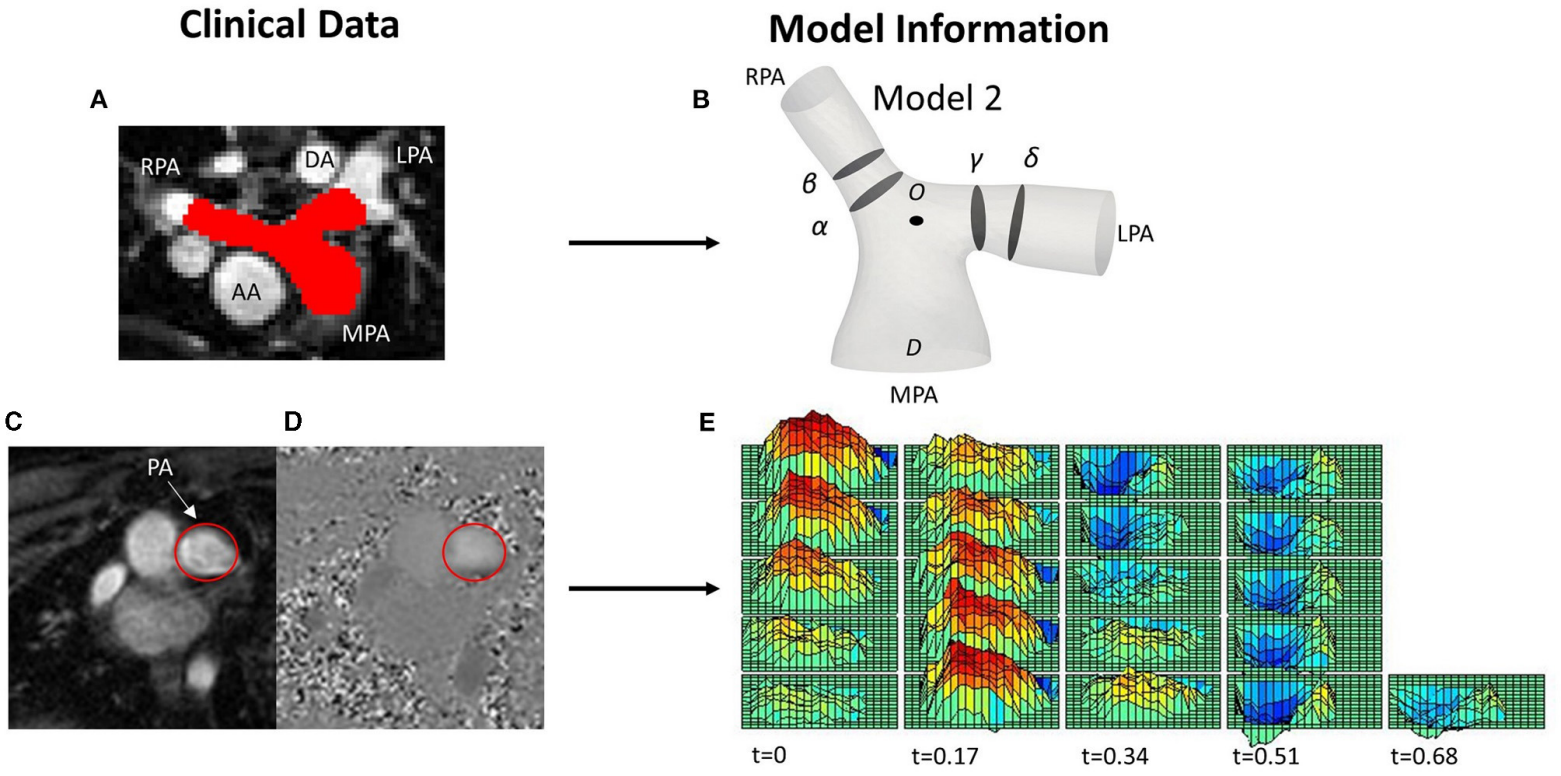
**(A)** MRI image with red color depicting the pulmonary arteries (PAs), including the main (MPA), left (LPA) and right pulmonary artery (RPA), and showing the descending (DA) and ascending aorta (AA) relevant to the PAs, for reference. **(B)** One of the reconstructed patient-specific models (Model 2), with slices (α) and (β) over the RPA, and (γ) and (δ) over the LPA shown in the model. **(C,D)** PC-MRI data from the same patient used for the extraction of the velocity profile at the MPA root, with the MPA encircled in red; **(C)** magnitude and **(D)** phase contrast (PC-MRI) image. **(E)** 3D-velocity profile extracted from PC-MRI data displaying the variation of the velocity both in space and time over a cardiac cycle.

Retrospective clinical data from seven patients with rTOF were used to study the blood flow in models of the pulmonary bifurcation. Demographic information about each patient is reported in Table 1, with an average age of the population being 26.3 ± 15.7 and the grade of regurgitation fraction varying from moderate to severe. Images of the pulmonary trunk of these patients, used for model reconstruction (Figures 1A,B), were acquired between 2012 and 2017 with a Siemens Avanto 1.5-Tesla MRI scanner (Siemens Healthcare, Erlangen, Germany), using the protocol “3D and Phase contrast” (TE = 2.08 or 2.18 ms, TR = 8.01–32.04 ms, FOV = 240–450 × 250–450 mm, Pixel resolution 192–256 × 192–256, Pixel spacing [1.2500;1.2500] – [1.5625;1.5625]). The scans were acquired with both ECG and respiratory gating. The clinical data include part of the clinical assessment of patients in the Great Ormond Street Hospital for Children, London, UK.

**Table 1:** Demographic and hemodynamic data of the repaired TOF cases.

**Patient**	**Sex**	**Age at scan**	**PA RF (%)**	**Grade**	**Flow split (Q_**RPA**_:Q_**LPA**_)**
1	Male	5 years	45	Severe	55.3:44.7
2	Male	12 years	41	Severe	64.8:35.2
3	Male	19 years	30	Severe	55.5:44.4
4	Male	23 years	40	Moderate	63.9:36.1
5	Male	30 years	48	Severe	75.4:24.6
6	Female	41 years	50	Moderate	76.6:23.4
7	Female	54 years	50	Severe	45.7:54.3*

PA, pulmonary artery; RF, regurgitation fraction, Grade refers to the regurgitation fraction, *the flow split of patient 7 was calculated as described in the methodology (section Extraction of Flow Data).

### Extraction of Flow Data

**Figure 2:**
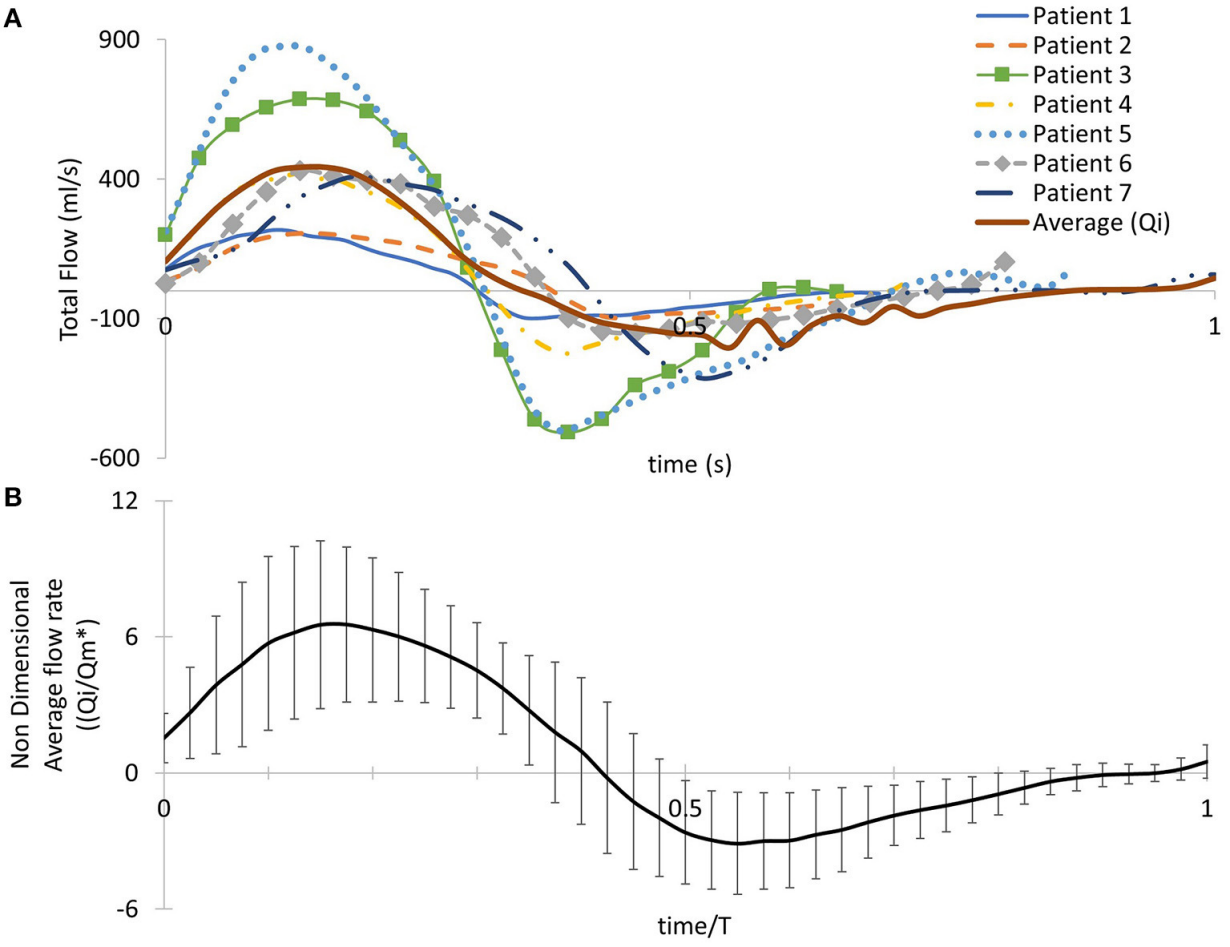
**(A)** Patient-specific pulsatile inlet flow rate waveform for all seven subjects and an averaged flow rate waveform (*Q*_*i*_) derived from the seven patient-specific waveforms. **(B)** The averaged flow rate waveform normalized with the mean value of the average flow rate over the cardiac cycle Qm*. Time was normalized with the period of the cardiac cycle of each patient. Error bars represent the standard deviation of the patient-specific flow waveforms from the average flow waveform.

To extract the 3D velocity profile at the MPA root from the phase-contrast MRI images (PC-MRI), the software Segment Research (24) for medical image analysis was used. Phase contrast and magnitude image stacks, over a cardiac cycle, were loaded as inputs for all geometries (Figures 1C,D). The lumen circumference of each MPA branch was marked on each frame of the cardiac cycle, and the diameter and flow rate profiles were exported for each stack of images for post-processing. The flow rate extracted at each time point was then divided by the area of the vessel at the specific time in order to consider the diameter changes during the cardiac cycle (9). The total inlet flow rate waveforms over the cardiac cycle are presented in Figure 2A for all seven patients. An average flow rate waveform, *Q*_*i*_, was calculated based on the seven flow waveforms after time was normalized with the period of the cardiac cycle of each patient (Figure 2A). This average flow waveform is also presented in Figure 2B normalized by the mean value of the average flow rate over each cardiac cycle Qm^*^. The average flow rates of each patient can be found in Table 2. In addition to the time-dependent flow rate waveforms, time, and spatially resolved 3D inlet velocity profiles were extracted for every time step of the cycle (Figure 1E).

**Table 2:** Diameters, mean flow rate, and mean and max velocities for the MPA, the RPA, and the LPA branches of each model.

**Model**	**D_**MPA**_**	**D_**RPA**_**	**D_**LPA**_**	**Q_**mean**_**	**Umean**	**Umean**	**Umean**	**Umax**	**Umax**	**Umax**
	**(m)**	**(m)**	**(m)**	**(ml/s)**	**_**MPA**_(m/s)**	**_**RPA**_(m/s)**	**_**LPA**_(m/s)**	**_**MPA**_(m/s)**	**_**RPA**_(m/s)**	**_**LPA**_(m/s)**
1	0.017	0.010	0.013	33.9	0.086	0.133	0.148	0.696	1.076	0.534
2	0.022	0.013	0.013	47.2	0.086	0.154	0.087	0.699	1.252	0.703
3	0.050	0.016	0.015	114.4	0.131	0.696	0.676	0.346	1.843	1.797
4	0.018	0.009	0.015	69.6	0.148	0.391	0.071	1.210	3.302	1.260
5	0.024	0.017	0.022	103.0	0.048	0.066	0.020	1.043	1.454	0.308
6	0.025	0.019	0.019	85.0	0.158	0.201	0.063	0.685	0.982	0.312
7	0.028	0.017	0.028	54.4	0.069	0.134	0.119	0.508	1.581	0.648

PC-MRI data were also available from the right and left pulmonary branches, except for patient 7 which lacked this information, and were used to calculate the flow split in each model, based on the net volume (forward minus backward volume, in ml, over the cardiac cycle) retrieved from Segment Research (Table 1). For patient 7, we used the information available from the other six patients to calculate the flow split, based on the following formula:


(1) $$\begin{array}{l} \frac{Q_{RPA-7}}{Q_{LPA-7}}=\frac{Q_{RPA-av}}{Q_{LPA-av}}\cdot {\left(\frac{R_{RPA-7}}{R_{LPA-7}}\cdot \frac{R_{LPA-av}}{R_{RPA-av}}\right)}^{2} \end{array}$$

where $\frac{Q_{RPA-av}}{Q_{LPA-av}}$ is the ratio of the average flow rate for the RPA and LPA branches of the six patients, and ${\left(\frac{R_{LPA-av}}{R_{RPA-av}}\right)}^{2}$, the ratio of the average radii of the LPA and the RPA squared. The result indicates a flow split *Q*_*RPA*_:*Q*_*LPA*_ of 45.7:54.3%, which is in accordance with the flow split reported in the clinical record for the specific patient. The average flow split based on the six patients with available patient specific data was found ~65.3:34.7% (*Q*_*RPA*−*av*_:*Q*_*LPA*−*av*_).

### Reconstruction of Patient-Specific Models

Clinical whole heart MRI images were used to segment the patient-specific three-dimensional structures using the open-source software ITK-SNAP (www.itksnap.org) (Figure 1A) (25). Initially, a semi-automatic active contour segmentation tool was used, followed by manual segmentation of the pulmonary trunk in each slice of the datasets to refine the process. The segmentation was extended until the first daughter branch on the right and left pulmonary branches. The geometries were then exported as surface mesh, in the stereolithography (STL) format. Smoothing was necessary to remove artifacts due to the image resolution and segmentation processes, while extensions were added in the model branches to avoid any effects from the boundaries. An extension of 0.5*D* was also added at the MPA inlet, where *D* is the diameter of the MPA inlet for each model. Two cross-sections of the lumen were taken on each branch of the PAs (Figure 1B). Cross-sections (α) and (γ) were taken at 0.4*D* and sections (β) and (δ) at 0.6*D* from the bifurcation origin, denoted as O. Point O is defined as the point where the branch splitting occurs, according to the branch splitting function of the open-source software VMTK (www.vmtk.org).

### Geometry Characterization

A geometric analysis of the models was conducted in the open-source software VMTK to identify parameters, including: the curvature of the RPA and LPA branches, torsion, tortuosity, the minimum inscribed sphere radius along the daughter branches, and in-plane and out-of-plane angles.

First, the centrelines c(s) of the models were generated, where (s) is the curvilinear abscissa (26). Curvature and torsion measure, respectively, the deviation of a curve from a straight line and its divergence from lying on the oscillating plane, computed as (24):


(2) $$\begin{array}{l} \kappa \left(s\right)=\frac{\left||c^{\prime }\left(s\right)\times c^{\prime \prime }\left(s\right)|\right|}{||c^{\prime }\left(s\right)|{|}^{3}},\;\tau \left(s\right)=\frac{\left[c^{\prime }\left(s\right)\times c^{\prime \prime }\left(s\right)\right]\cdot \;c^{\prime \prime \prime }\left(s\right)}{\left||c^{\prime }\left(s\right)\times c\left(s\right)|\right|} \end{array}$$

The curvature and torsion were measured in 1/mm and evaluated along the centreline on a curvilinear abscissa, normalized by the distance corresponding to the peak curvature value closer to the bifurcating branches. Therefore, the value of 1.0 in the normalized curvilinear abscissa represents the location of peak curvature for each branch. Error bars were used to signify the deviation of the patient-specific values from the calculated averages.

**Figure 3:**
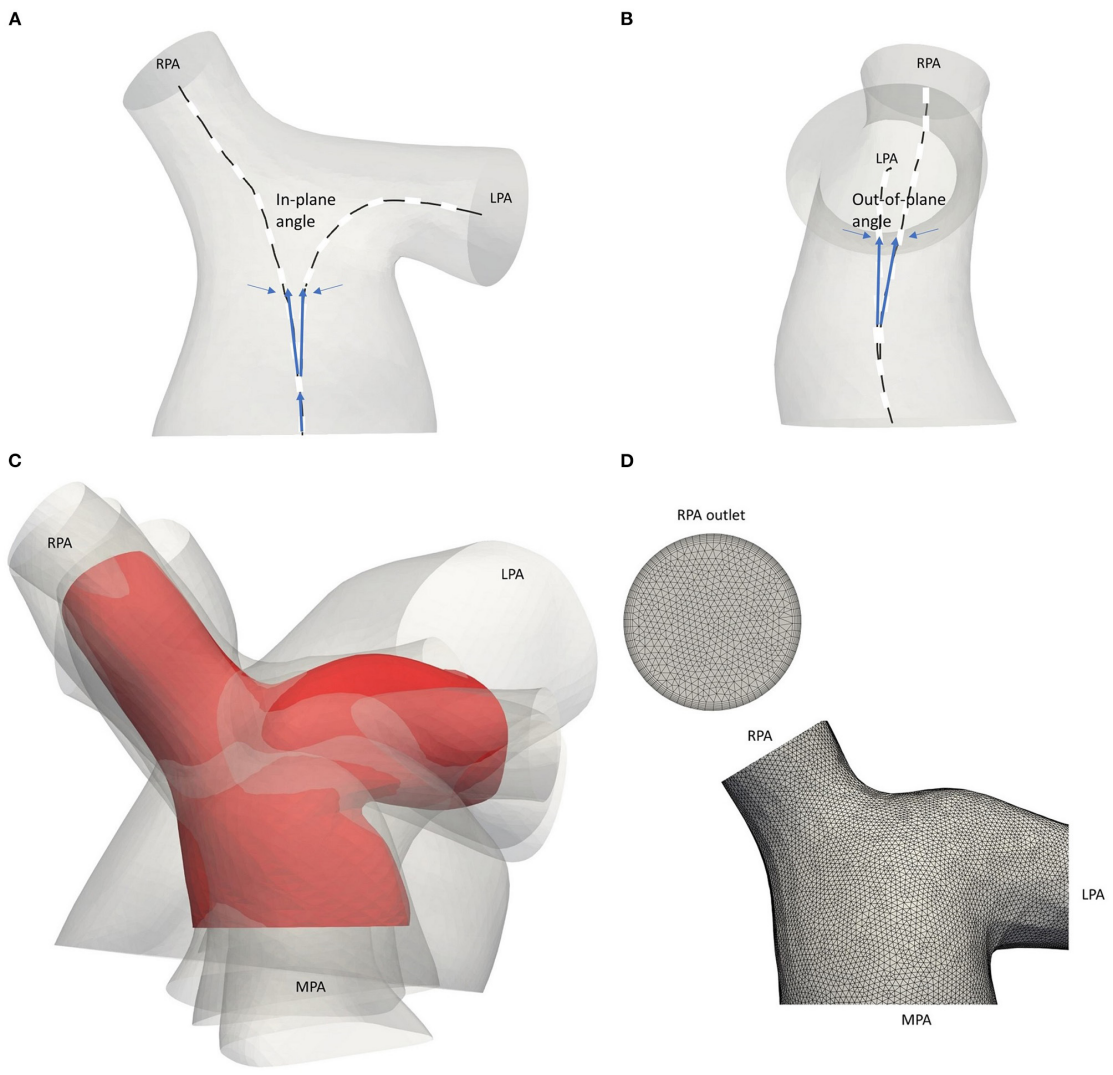
Schematic representation of the **(A)** in-plane and **(B)** out-of-plane angles. **(C)** Average geometry of the five adult TOF patients (red) with the patient-specific models shown transparent. Models are co-registered based on point O of Figure 1B. **(D)** Computational mesh of the average geometry around the pulmonary junction. The boundary layer at the RPA outlet is also displayed.

Tortuosity describes the relative increment in the length of the curve from a straight line and was calculated by *x* = *L/D – 1*, where *L* is the length of the centreline and *D* the Euclidean distance between its endpoints (27). The maximal inscribed sphere radius measures the radius of the vessel locally and by identifying the minimum radius along the centrelines, stenotic regions can be assessed (28). The in-plane and out-of-plane angles were calculated based on the bifurcation plane, defined by points along the centreline of the model, providing information on the bifurcation angle and planarity, respectively (Figures 3A,B) (29). The in-plane angle is presented with respect to the angle formed between the MPA and each daughter branch and, therefore, takes only positive values. A positive sign of the out-of-plane angle is related to the clockwise rotation of the branch.

### Anatomical Average Geometry

An anatomical average geometry was created using the statistical shape analysis software Deformetrica (https://www.deformetrica.org/) (30) based on a forward approach as described by Bruse et al. (31). Only the adult patients of this study were considered to compute the anatomical average geometry; the models of patients 3–7 were registered with the model from the 6th patient, also assumed as the reference model, and using point O (Figure 1B) as the common origin for the alignment. To account for the initial assumption of the reference geometry, the process was repeated six more times, utilizing the computed template as the reference geometry in every following iteration of the process. The anatomical average geometry used for the flow simulations was decided based on the distance between the surfaces of the reference and the calculated template on each repetition, and until the maximum surface distance reached 1 mm, which accounts for ~1% of difference (Figure 3C). A template kernel width of 15 and a deformation kernel width of 7 were used for the computations; the kernel width controls the length of the deformations allowed and the larger its value, the more global shape variations the atlas captures. Nevertheless, it is found that no fine parameter tuning is needed as the atlas is stable for a large range of values (32).

### Numerical Simulations

The methodology followed to set the numerical simulations is described in the next sections, including mesh generation, boundary conditions (BCs) setup, and computational approximation.

### Computational Mesh

The computational mesh was generated using the commercial software ANSA v20.0 (BETA CAE Systems, www.beta-cae.com/ansa.htm). The volume mesh consisted primarily of tetrahedra elements and a boundary layer mesh composed of seven layers of prismatic cells was added, with the first layer at a distance of 0.005 cm away from the wall (Figure 3D). The y^+^ value was calculated for the average geometry following the same methodology described in Boumpouli et al. (23), and its mean value over a cardiac cycle was 0.074. The same computational mesh strategy was adopted for all models. According to a grid independence analysis, where the integral of the wall shear stress on a cross-section of the pulmonary bifurcation was measured, the total number of elements was set around 2.5 million for each geometry.

### Mapping of 3D Velocity Profile

An in-house python code (https://github.com/emisau/3DFlowProfile_MRI2CFD) was used for mapping the spatially-varying velocity profiles of the patients, extracted from the PC-MRI data, to the inlet of each patient-specific mesh. The code takes two inputs. The first is a comma-separated value (CSV) file exported from the Segment Research software package. It stores spatial coordinates, time in the cardiac cycle and velocity magnitude information for each voxel of a PC-MRI image. The second input file is a triangulation of the inlet surface of the fluid domain holding coordinates and connectivity information for each element of that mesh. The latter file is stored in ascii format (^*^.msh) exported from the open-source mesh generator Gmsh (33). The main idea of the code is to transfer the flow profile information from the voxel grid to the inlet surface triangulation by overlaying them in a common coordinate system. A polar coordinate transformation is applied to both grids. Subsequently, a closest node search algorithm is used to obtain spatial correspondence between the grids. Finally, flow information from the voxel grid is mapped onto the mesh grid. The orientation of the 3D velocity profile was assumed aligned with the inlet of each model and was provisionally checked using anatomical landmarks in the MRI and PC-MRI images. The output of the code is a series of text files containing velocity and coordinates information, one for each time step, that can be directly read by the numerical solver to set the inlet flow condition of the fluid domain.

### Computational Model

Flow simulations were carried out in the open-source CFD software OpenFOAM v.1812 (https://www.openfoam.com/) and blood was considered as a Newtonian, incompressible fluid. The Reynolds Averaged Navier–Stokes equations for turbulent flows were used (34):


(3) $$\begin{array}{ll} \nabla \cdot \bar{u} & =0 \end{array}$$

(4) $$\begin{array}{ll} \rho \frac{\partial \bar{u}}{\partial t}+\rho \nabla \cdot \left(\bar{uu}\right) & =-\nabla \bar{p}+\nabla \cdot \mu \left(\frac{\partial {\bar{u}}_{i}}{\partial x_{j}}+\frac{\partial {\bar{u}}_{j}}{\partial x_{i}}\right) \end{array}$$

where $\bar{u}$ is the mean velocity field and *p* is the pressure. Blood density, ρ, and the dynamic viscosity, μ, were set to 1,060 kg/m^3^ and 0.004 Pa s, respectively. The *k*–ω shear stress transport (SST) turbulence model was assumed, that utilizes two differential transport equations, one for the turbulent kinetic energy *k*, and one for the dissipation rate, ω, as described in Menter et al. (35), and with the following model specifications: turbulent intensity (***I*** **=** 0.16^*^Re^**−1/8**^), energy [***k*** **=** 3/2^*^(UI)^**2**^], length scale (***L*** **=** 0.07^*^D), and specific dissipation rate (**ω** **=** $\sqrt{k}$/0.09^*^L), where *Re* is the Reynolds number, *U* is the mean velocity.

Two sets of simulations were performed. In the first set, the uniform pulsatile (time—but not space dependent) average flow waveform (described in section Extraction of Flow Data, Figure 2A) was specified at the inlet of all seven models, including the anatomical average geometry, and the average flow split of ~65.3:34.7% (*Q*_*RPA*_:*Q*_*LPA*_) was applied at the branch outlets. The assumption of the same BCs on all geometries allows the identification of differences in the flow patterns due to geometrical variations. In the second set of simulations, the patient-specific boundary conditions were used with the corresponding patient model. 3D velocity profiles (time- and space-dependent Figure 1E) were assigned at all geometry inlets. At the outlets of the models, the flow splits presented in Table 1 were specified. In the LPA and RPA outlets, the flow splits in both sets of simulations were imposed by specifying the transient flow rate in each branch, and velocity was uniform and only time varying. The walls of all geometries were assumed rigid, and the no-slip boundary condition was assigned. All numerical simulations were performed with the pisoFoam solver, of the OpenFOAM® open-source library, for transient incompressible, turbulent flow, using the pressure-implicit with splitting operators (PISO) algorithm and the second order bounded Gauss linear upwind divergence scheme, the backward differential scheme for the time discretization and the Gauss linear gradient numerical scheme were utilized.

The mean Reynolds (*Re*), Womersley (*Wo*), and Dean (*De*) numbers for all geometries were calculated based on the following formulas:


(5) $$\begin{array}{l} Re=\frac{UD}{\nu } \end{array}$$

(6) $$\begin{array}{l} Wo=\frac{D}{2}\sqrt{\frac{2\pi }{\nu T}} \end{array}$$

(7) $$\begin{array}{l} De_{\max}=Re_{\max}\sqrt{\frac{D}{2R}} \end{array}$$

where ν is the kinematic viscosity and *R* is the radius of curvature (1/curvature). Re_max_ was calculated using the local velocity at each branch, while to calculate the Dean number in the RPA and LPA branches, *Re*_*max*_, as calculated for the MPA branch, was multiplied by the percentage of flow split in Equation (7), as suggested by another study in the literature (11). The Reynolds number, *Re*, is used to characterize whether the fluid presents a laminar or turbulent flow behavior, based on the ratio of inertial to viscous forces, while the Womersley, *Wo*, and Dean numbers are dimensionless parameters utilized to characterize flow pulsatility in relation to viscous flows and flows in curved pipes, respectively. To calculate the *Re* at the inlet and the outlets of the models, the diameter at each boundary was considered. A table with the diameters, the mean flow rate, the mean and the max velocity of all three branches for each model is provided (Table 2). The time averaged wall shear stress (TAWSS) was also calculated for all models, using the formula $TAWSS=\frac{1}{T}\int_{0}^{T}\tau_{\omega }dt$, where τ_ω_ is the wall shear stress and *T* is period of the cardiac cycle. TAWSS was normalized with the value of the TAWSS as calculated at the inlet of the models and presented in Supplementary Table S3.

### Study Sensitivity

In an attempt to confirm the methodology followed in this study, the effect of various parameters was investigated. Firstly, different smoothing factors were considered in model 1 at the reconstruction stage of the process (section Reconstruction of Patient-Specific Models), and the wall shear stress distribution in the models were compared. The comparison indicated that doubling the smoothing factor in the model, did not alter the development of flow and therefore, the higher smoothing factor was adapted in this study for all models. In addition, the point from which the left and the right pulmonary arterial branches were extended was altered in model 4 and a difference of 5.4% was found on the integral of the wall shear stress values developed around the pulmonary junction. Moreover, a comparison between the 3D velocity profile and a pulsatile waveform with a plug profile, was conducted in model 1, and higher shear stress areas were apparent when the 3D velocity profile was assigned at the inlet of the models. The difference was evaluated for slices (α) and (γ) of Figure 1B, and was about 10% for the RPA, and ~19% for the LPA branch.

### Supplementary Material

Further analysis of the results, including the min/mean/max torsion calculated in the outlet branches of the models, secondary flow visualized by in-plane velocity vectors in the RPA and LPA cross-sections of all models, and the oscillatory shear index distribution, can be found in the Supplementary Material. In addition, the values used for the non-dimensionalization of the TAWSS and the percentage of difference between the average and patient-specific values for the flow splits and the inlet flow are reported.

## Results

**Figure 4:**
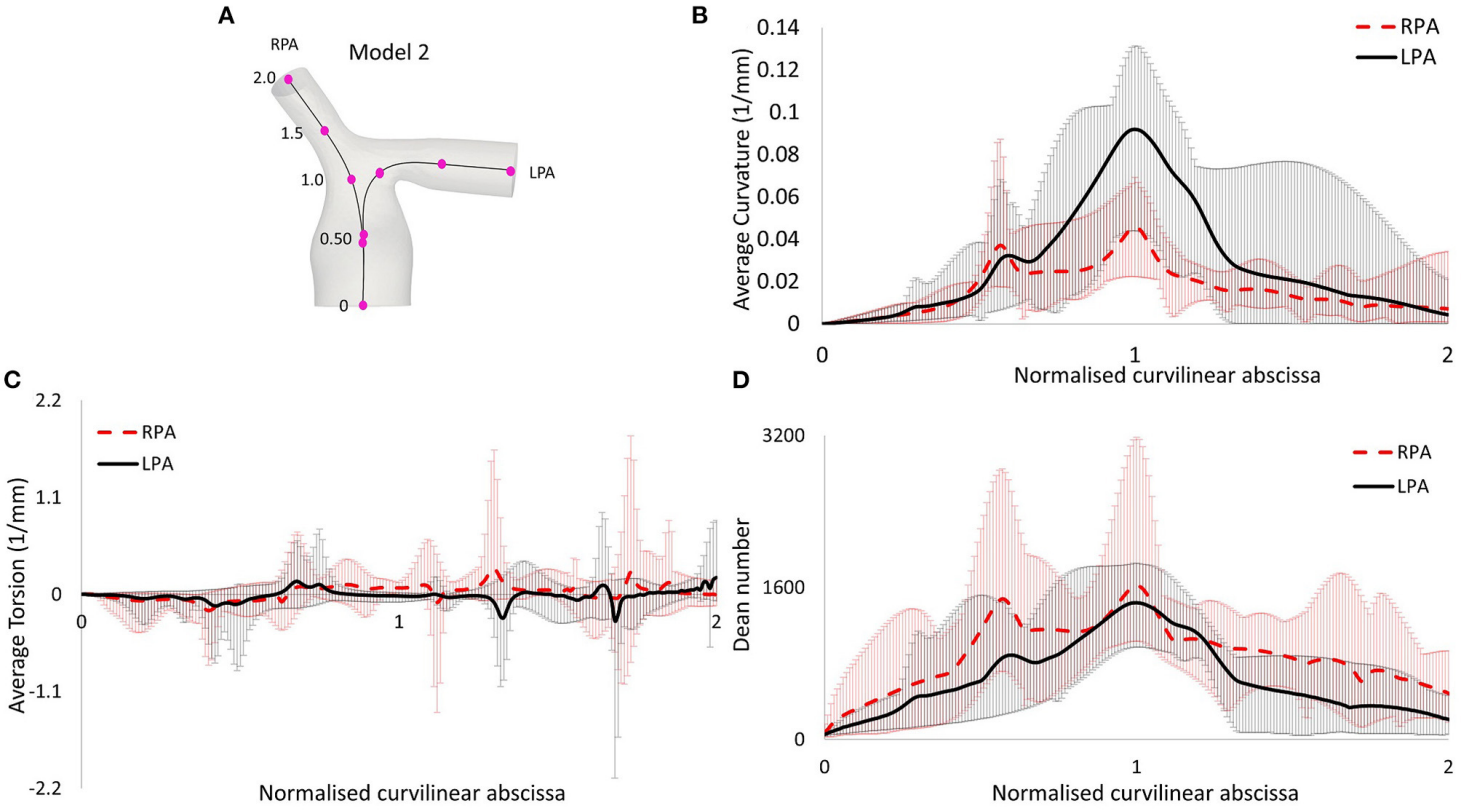
**(A)** Centerlines along the LPA and RPA of model 2, with points indicating the curvilinear abscissa, normalized by the distance corresponding to the peak curvature value closer to the bifurcating branches. Therefore, the value of 1.0 represents the location of peak curvature for each branch. **(B)** Average curvature plot. **(C)** Average torsion plot, and **(D)** Average Dean number plot, with *x*-axis indicating distance from point O.

**Figure 5:**
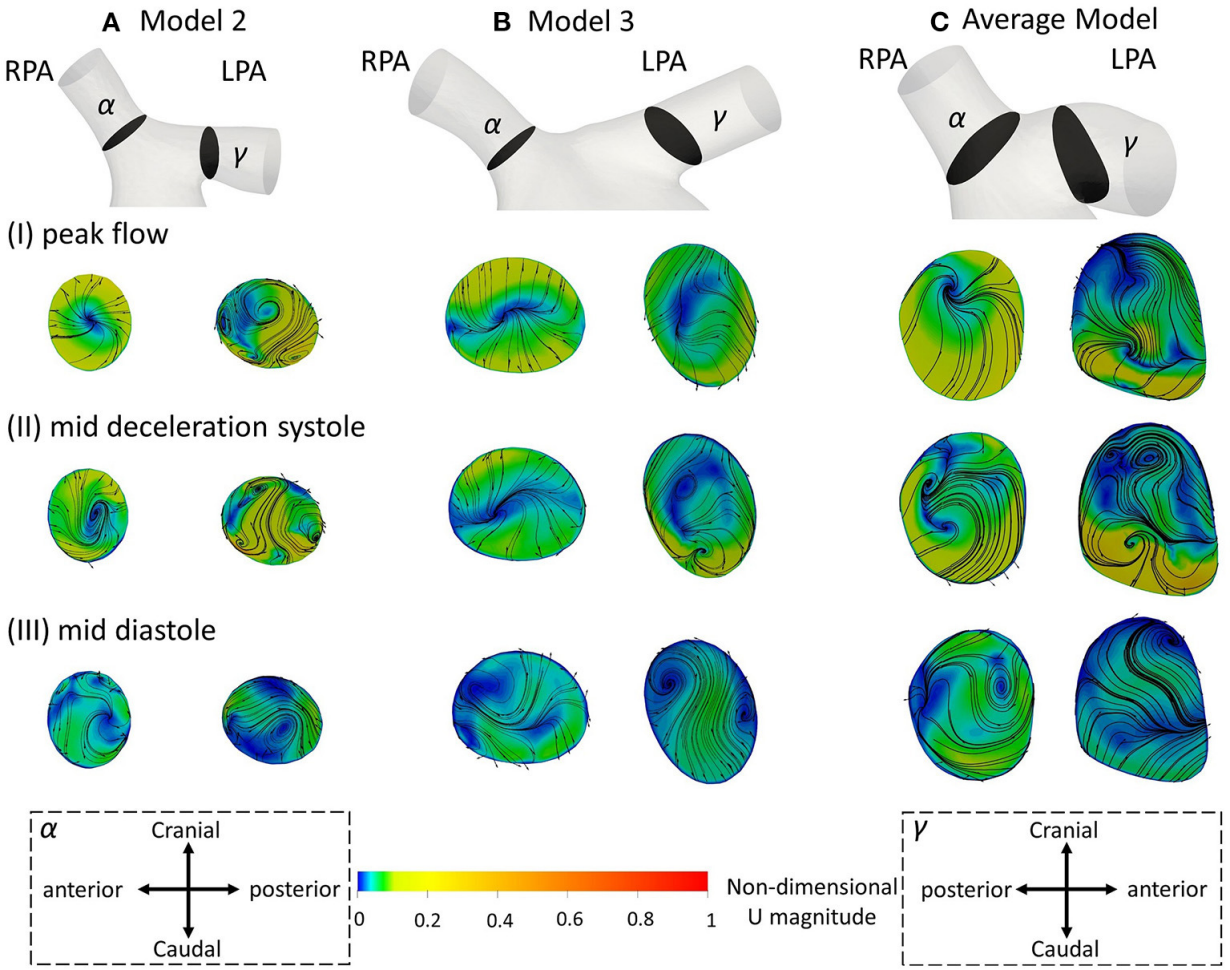
Secondary flow visualized by in plane velocity vectors and contours of normalized velocity normal to the slice during (I) peak flow; (II) mid deceleration at systole; and (III) mid diastole, for **(A)** model 2; **(B)** model 3; and **(C)** the average model. Non-dimensionalization was performed by division with the maximum velocity of each patient during the cardiac cycle. Points where slices (α) and (γ) are taken are visible in Figure 1B. Cross-sections are oriented with the top and the bottom edges corresponding to the cranial and caudal positions, respectively and left and right to the anterior and posterior of the pulmonary artery, for the RPA, and to the posterior and anterior of the pulmonary artery, for the LPA, respectively. Cross-sections are in scale.

**Figure 6:**
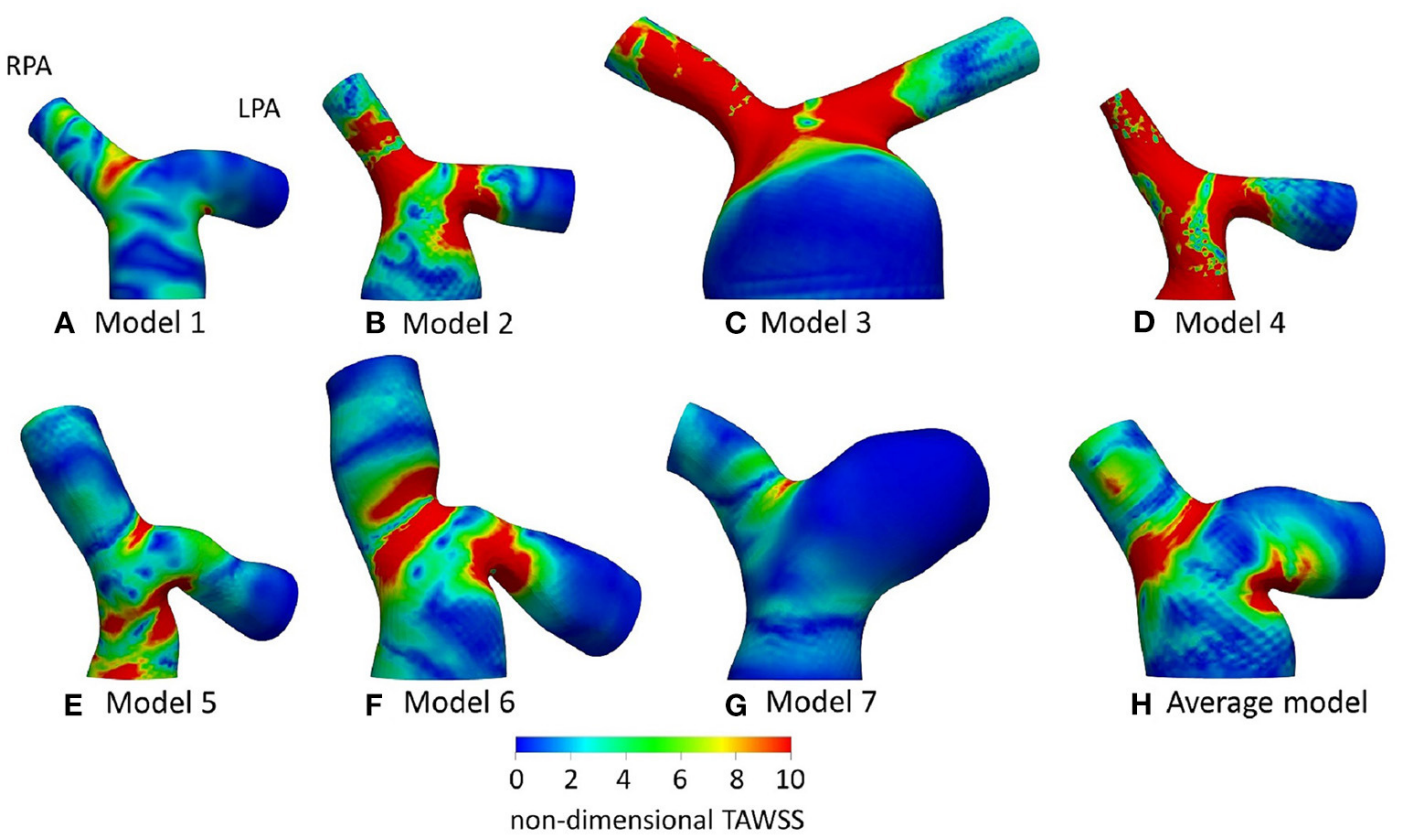
Non-dimensionalized time-averaged wall shear stress (TAWSS_n_) distribution, normalized with the corresponding value at the inlet of each models, for **(A)** Model 1; **(B)** Model 2; **(C)** Model 3; **(D)** Model 4; **(E)** Model 5; **(F)** Model 6; **(G)** Model 7; and **(H)** Average model. Averaged boundary conditions are used at the inlet and the outlets of all models.

**Figure 7:**
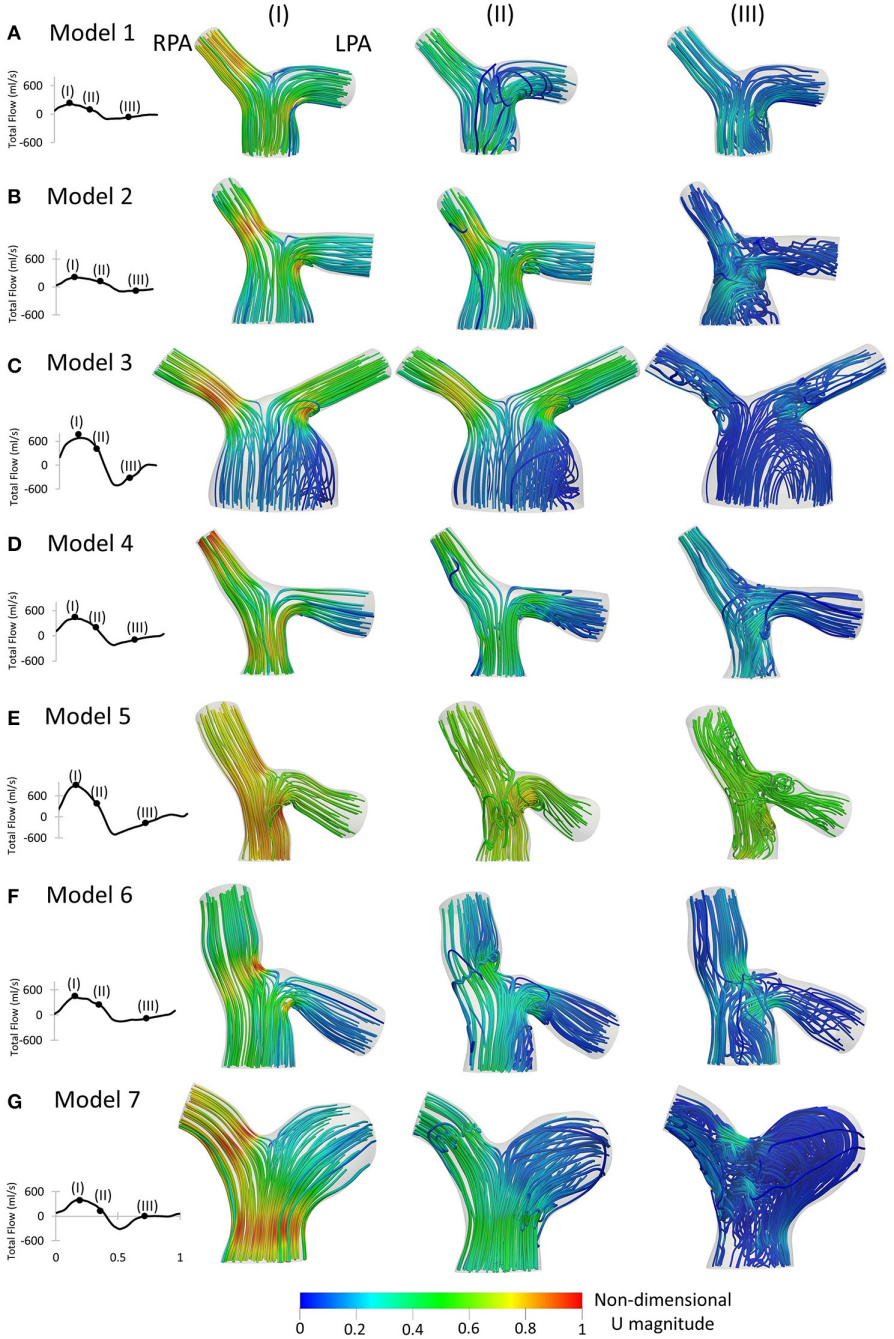
Left to Right: Patient-specific flow waveforms and velocity streamlines at (I) peak flow; (II) mid deceleration at systole; and (III) mid diastole, for **(A)** Model 1; **(B)** Model 2; **(C)** Model 3; **(D)** Model 4; **(E)** Model 5; **(F)** Model 6; and **(G)** Model 7. Streamlines are colored by non-dimensionalized velocity magnitude based on the maximum velocity during the cardiac cycle of each patient. The RPA and the LPA branches are indicated in model 1.

**Figure 8:**
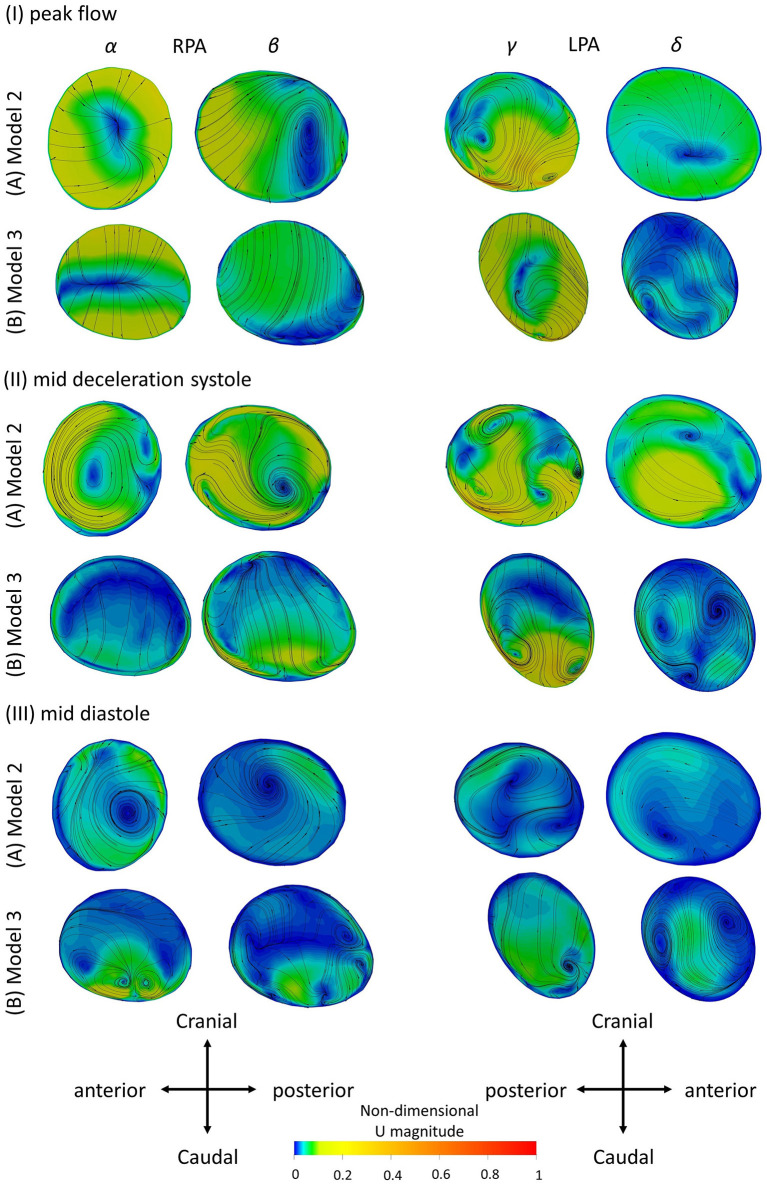
Secondary flow visualized by in plane velocity vectors and contours of normalized velocity normal to the slice during (I) peak flow; (II) mid deceleration at systole; and (III) mid diastole, for **(A)** Model 2; and **(B)** Model 3. Non-dimensionalization was performed by division with the maximum velocity of each patient during the cardiac cycle. Points where slices (α) to (δ) are taken are visible in Figure 1B. Cross-sections are oriented with the top and the bottom edges corresponding to the cranial and caudal positions, respectively and left and right to the anterior and posterior of the pulmonary artery, for the RPA, and to the posterior and anterior of the pulmonary artery, for the LPA, respectively. Cross-sections are in scale.

**Figure 9:**
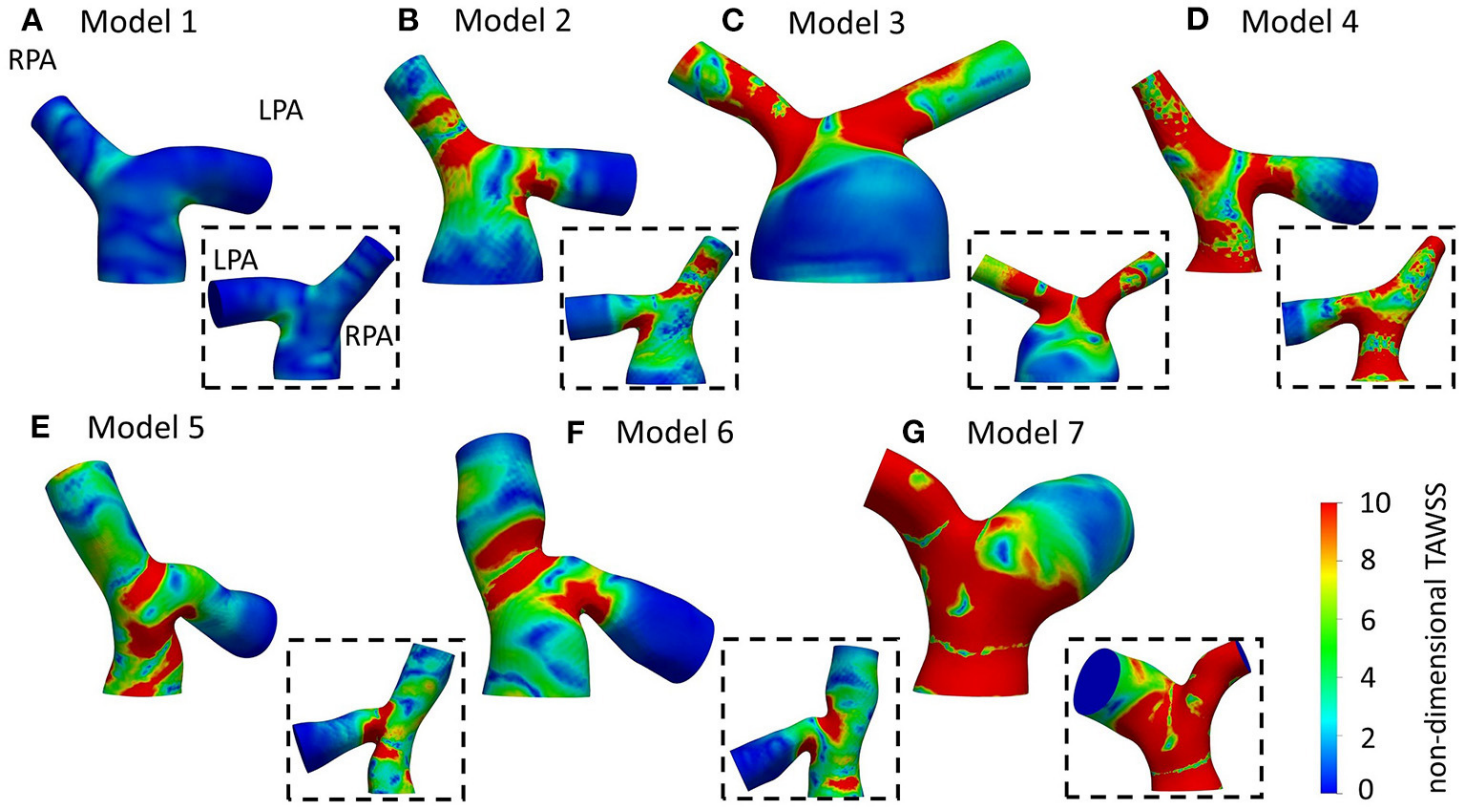
Non-dimensionalized time-averaged wall shear stress (TAWSS_n_) distribution, normalized by the wall shear stress value at the inlet of each model, respectively, for **(A)** Model 1; **(B)** Model 2; **(C)** Model 3; **(D)** Model 4; **(E)** Model 5; **(F)** Model 6; and **(G)** Model 7. Insets show the back view of the models. The LPA and RPA branches are indicated in Model 1.

**Figure 10:**
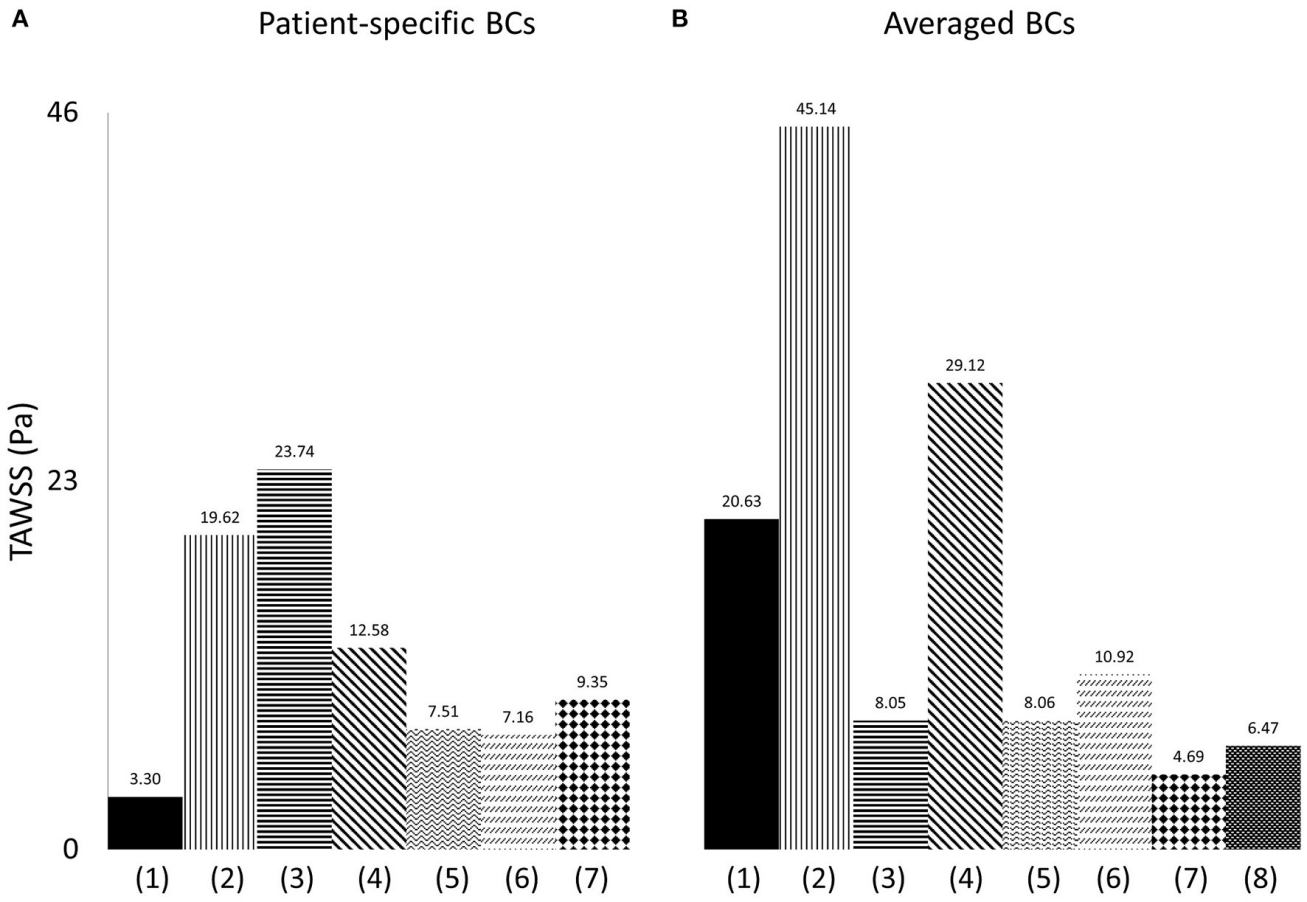
Time-averaged wall shear stress (TAWSS) plot, derived from the TAWSS values of eight points located along the perimeter of cross-sections (α) and (γ), and presented in Pascal (Pa), when **(A)** patient-specific, and **(B)** averaged boundary conditions are specified, and for (1) model 1; (2) model 2; (3) model 3; (4) model 4; (5) model 5; (6) model 6; (7) model 7; (8) the average model.

In the following paragraphs, the effect of the morphology and flow characteristics are presented in a series of figures and tables. The three main sections in which the results are separated are the geometry characterization (Table 3 and Figure 4), the flow characterization (Table 4) and the computational analysis, which is further divided on the averaged boundary conditions (Figures 5, 6) and patient-specific boundary conditions (Figures 7–10).

**Table 3:** Geometric analysis of the patient-specific models: curvature, tortuosity, minimum inscribed sphere radius along the daughter branches, and in-plane and out-of-plane angles.

**Model**	**Curvature RPA (mm^**−1**^) (mean/max)**	**Curvature LPA (mm^**−1**^) (mean/max)**	**Tortuosity (RPA/LPA)**	**Min sphere radius (mm) (RPA/LPA)**	**In-plane angles (RPA/LPA)**	**Out-of-plane angles (RPA/LPA)**
1	0.021/0.066	0.036/0.103	0.017/0.115	4.8/5.8	132.1°/118.7°	1.3°/27°
2	0.015/0.036	0.029/0.110	0.017/0.132	4.5/4.8	142.6°/119.9°	9.4°/−21.4°
3	0.014/0.087	0.012/0.068	0.044/0.091	5.7/4.8	163.8°/168.2°	7.1°/−3.8°
4	0.018/0.035	0.036/0.105	0.013/0.121	3.5/4.1	142.2°/110.1°	−15.4°/11.2°
5	0.014/0.053	0.032/0.131	0.011/0.258	7.0/6.5	135.9°/118.6°	−35.1°/58.9°
6	0.016/0.034	0.042/0.094	0.003/0.182	6.3/5.3	160.3°/126.7°	−26.1°/31.5°
7	0.019/0.046	0.015/0.058	0.103/0.111	6.7/10.8	124.4°/160.1°	−12.5°/16.9°
Mean value	0.017/0.051	0.029/0.096	0.030/0.144	5.5/6.0	143.0°/131.8°	−10.2°/17.2°

**Table 4:** Mean and maximum Reynolds (Re), Womersley (Wo), and Dean number (De).

**Model**	**Re_**mean_MPA**_ (Re_**max_MPA**_)**	**Re_**mean_RPA**_ (Re_**max_RPA**_)**	**Re_**mean_LPA**_ (Re_**max_LPA**_)**	**Wo**	**De_**max_RPA**_**	**De_**max_LPA**_**
1	397 (3,199)	367 (2,958)	520 (1,873)	13.6	1,034	1,178
2	504 (4,093)	542 (4,408)	300 (2,434)	17.3	1,302	1,215
3	1,722 (4,561)	2,962 (7,846)	2,615 (6,925)	40.3	2,115	1,450
4	696 (5,694)	905 (7,638)	290 (5,127)	13.6	1,412	1,844
5	297 (6,529)	305 (6,695)	116 (1,760)	16.3	3,180	1,851
6	1,023 (4,450)	1,011 (4,936)	315 (1,537)	17.7	1,934	972
7	521 (3,823)	718 (6,918)	886 (4,807)	18.3	1,085	1,854
Mean value	737 (4,621)	973 (5,914)	720 (3,495)	19.6	1,723	1,481

### Geometry Characterization

The morphological features described on section Reconstruction of Patient-Specific Models of the methodology are listed in Table 3. In the majority of the models, the curvature of the LPA branch was higher compared to the RPA (Table 3; Figures 4A,B), with the highest mean curvature of 0.042 mm^−1^ in the LPA of patient 6. Tortuosity was higher in the LPA branch of all models, and the largest value of 0.258 was observed in the LPA of patient 5. The minimum sphere radius was smaller on average in the RPA branch. Curvature in the LPA branch increased on average along the centrelines of all patients (Figure 4B) until it reached a peak approximately at the entrance of the daughter branches, before decreasing. Two peaks were observed in the average torsion plots (Figure 4C) for both the RPA and the LPA further downstream along the daughter branches. For the RPA, the peaks were positive, indicating a counter-clockwise rotation of the osculating circle, whilst they were negative for the LPA, signifying the clockwise shift of the branch. Nevertheless, a mean torsion close to zero is observed along the centrelines of both branches.

The in-plane bifurcating angle of the RPA (Table 3) was in most cases bigger compared to the LPA, indicating a more acute angle between the MPA and the LPA. Exception to this were patients 3 and 7, where in 3 the two branches had very similar bifurcating angles. The average in-plane angles for the RPA and LPA branches were, respectively, 143.0° and 131.8°. The out-of-plane angles suggest that the LPA deviated more from the bifurcation plane. In the majority of models, the rotation of the LPA was clockwise, while in four out of seven patients the rotation was anti-clockwise for the RPA branch. The mean value of the out-of-plane angles as calculated for the RPA and LPA branches were −10.2° and 17.2°, respectively (Table 3).

### Flow Characteristics

Following Equations (5–7), the Reynolds (mean and maximum), Womersley and Dean numbers were calculated for all models and are reported in Table 4. The mean *Re* varied during the cardiac cycle of the seven cases between 297 to 1,722 and, therefore, different flow patterns were expected during the cardiac cycle. The average calculated *Re*_*mean*_ values were 737 for the MPA, 973 for the RPA and 720 for the LPA, therefore, the highest *Re* was observed in the RPA branch. *Wo* was in the range of 13.6–40.3, as calculated at the inlet of the models for the seven cases examined, indicating a large frequency of pulsations (36), and with the highest value found in model 3. Finally, the average Dean number calculated for the RPA (*De*_*max*_*RPA*_ = 1,723) was higher compared to the LPA (*De*_*max*_*LPA*_ = 1,481). The Dean plots for the two branches (Figure 4D), indicate a similar increase as with the curvature plots, with the maximum Dean number located approximately at the entrance of the daughter branches.

### Computational Analysis

In the following paragraphs, we follow a top-down analysis by examining first the use of averaged boundary conditions on the anatomically average and personalized geometries (effect of geometry, section Averaged Boundary Conditions) and, then, the application of patient-specific boundary conditions on individualized geometries (patient-specific effects, section Patient-Specific Boundary Conditions).

### Averaged Boundary Conditions

Initially, the average flow waveform of Figure 2A and the average flow split (section Extraction of Flow Data) were used as the boundary conditions in the patient-specific models and the anatomical average geometry in order to better understand the effect of geometry in the flow development in the pulmonary arteries (Figures 5, 6). Although the anatomical average geometry was derived from a small number of TOF patients and cannot be considered representative of the population, its purpose is to help clarify the importance of patient-specificity in the models.

Figure 5 displays the secondary flow in cross-sections (α) and (γ) along the RPA and LPA, as defined in Figure 1B, in models 2, 3 and the average geometry, using averaged BCs, at three different time points: (I) peak systole, (II) mid deceleration at systole, and (III) mid diastole. Models 2 and 3 were chosen because the patients are of similar age and with a severe grade of regurgitation fraction, but have different flow characteristics (Table 3), which could facilitate the comparison between observed differences. During peak flow, vortices appeared in the LPA of models 2 [Figure 5AI (γ)], while no vortices were visible in the LPA of model 3 and the average model, or any RPA branch (Figure 5I). Progressing to mid deceleration at systole, the LPA branches of all models displayed counter-rotating vortices [Figure 5II (γ)]. For the RPA branches, a stable focus was visible in model 2 and the average model, characterized with velocity vectors toward the center of the vortex, according to Perry and Steiner's (37). Finally, at mid diastole, more complex flow patterns were observed in both the RPA and LPA branches of the models (Figure 5III). A more detailed description of the secondary flow can be found in the Supplementary Material.

TAWSS distribution, normalized by the value at the inlet of each model (denoted as TAWSS_n_), is presented in Figure 6 for all models. In general, higher wall shear stresses were observed at the entrance of the LPA and the RPA branches, while lower TAWSS_n_ values were visible further downstream at the LPA branch. In model 4 (Figure 6D), high TAWSS_n_ was also developed along the MPA branch. A small region of low TAWSS_n_ was found at the bifurcating wall in all models, indicating the stagnation point, where flow impinged before splitting and entering the daughter branches. Model 3 (Figure 6C) had an extensive area of low shear stress values in the enlarged MPA, while the lowest non-dimensionalized TAWSS values were found in model 7 (Figure 6G). The average model (Figure 6H) captured some main characteristics of the TAWSS patterns of models 1, 2, 3, and 6 (Figures 6A–C,F), particularly the localization of high TAWSS_n_ regions at the entrance of the branches and lower values near the flow divider.

### Patient-Specific Boundary Conditions

In the following subsections, computational results from matching patient-specific boundary conditions with their respective geometry are presented, analyzing velocity streamlines, secondary flow and the time-averaged wall shear stress distribution.

### Contours of Velocity and Velocity Streamlines

Velocity streamlines colored by non-dimensionalized velocity at three time points, peak flow (Figure 7I), mid deceleration during systole (Figure 7II), and mid diastole (Figure 7III), are examined. The patient-specific waveforms are also provided to indicate these time points. At peak systole, high velocities were developed at the entrance of the RPA and in the LPA opening, close to the MPA wall, in all models (Figures 7AI–GI). In models 5 and 7 (Figures 7EI,GI), and to a smaller extend in model 4 (Figure 7DI), high velocities were also visible along the MPA. A small recirculation zone was developed at the entrance of the LPA branches proximal to the MPA wall (Figures 7BI–FI), except in models 1 and 7 (Figures 7AI,GI).

During deceleration (Figure 7II), the recirculation zone at the entrance of the LPA of all models was enlarged and flow recirculation was also visible in the LPA of geometries 1 and 7 (Figures 7AII,GII). In models 3, 5, and 6 (Figures 7CII,EII,FII), flow separation occurred additionally within the MPA, while in the MPA of model 1 (Figure 7AII), a small area of reversed flow was observed. Along the RPA, complex flow was mostly observed for models 6 and 7 (Figures 7FII,GII).

Finally, during mid diastole (Figure 7III), complex flow patterns appeared in the pulmonary junction of the models. Highly disturbed flow was visible in models 2, 5, 6, and 7 (Figures 7BIII,EIII-GIII). In model 3 (Figure 7CIII), reverse flow was observed in the MPA and flow recirculation zones appeared in both the RPA and LPA. Only model 1 (Figure 7AIII) did not exhibit recirculation along the RPA and LPA branches.

### Secondary Flow

To investigate the secondary flow developed during the cardiac cycle, contours of velocity normal to the cross-sections of models 2 and 3 are presented in Figure 8, overlaid by the in-plane velocity vectors at (I) peak systole, (II) mid deceleration of systole and (III) mid diastole. Velocity values are presented non-dimensionalized with the corresponding maximum velocity developed in each model during the cycle.

During peak flow, vortices were visible in the LPA branch of both models, in cross-section (γ) [Figure 8I (γ)]. For the RPA branch two small vortices were formed further downstream along the branch [Figure 8I (β)]. In mid deceleration at systole, more complex flow patterns were observed. Counter-rotating vortices existed in the LPA of the branches in cross-sections (γ) were reduced in number further downstream for model 2 [Figure 8AII (δ)], but increased for model 3 [Figure 8BII (δ)]. Vortices were visible in the RPA of the models as well [Figure 8II (α, β)]. Finally, at mid diastole, counter-rotating vortices can be seen in all cross-sections of model 3 (Figure 8BIII), except (γ), while mostly stable foci are apparent in the cross-sections of model 2 (Figure 8AIII). A more detailed description of the secondary flow can be found in the Supplementary Material, along with analysis of the other models.

The results of the secondary flow pattens developed in the RPA of models 2 and 3 are comparable with those presented in Figure 5 during peak flow and mid deceleration at systole; some differences are visible in mid diastole where more foci were formed in the RPA of model 2 [Figure 5AIII (α)] and no vortices were observed in the RPA of model 3 [Figure 5BIII (α)] with the averaged boundary conditions. Similarities exist in the flow patterns developed in the LPA cross-sections as well. Differences were found in model 2, at peak flow, where two pairs of vortices were developed with the averaged boundary conditions [Figure 5AI (γ)]. For model 3, the flow patterns developed during mid deceleration at systole and mid diastole [Figures 5BII,III (γ)] resemble more those observed further downstream in Figure 8 [Figures 8BII,III (δ)].

The geometrical and flow parameters of models 2 and 3 (Tables 2, 3), indicate higher *Re* and *De* numbers in the RPA branch of both models, while the LPA branches demonstrate elevated tortuosity. The LPA of model 2 has higher curvature compared to the RPA, but the opposite is true for model 3. In general, more complex secondary flow patterns appeared in the LPA branches of the models, indicating that tortuosity has a greater impact in vortex formation. Furthermore, a comparison of these parameters between the two models, reveals that the RPA branch of model 3 has a higher maximum curvature and tortuosity, and higher *Re* and *De* numbers than those of model 2, while for the LPA branch, model 3 is characterized by higher *Re* and *De* numbers, but the curvature and tortuosity are higher in model 2. These results further highlight the impact of tortuosity, and also curvature in the secondary flow, as more vortices are noticed in the LPA branch of model 2. Finally, an increased number of vortices exists in the LPA of model 2 when the averaged BCs are used, however this difference could be explained by the considerable difference in the peak inlet flow between patient-specific and averaged velocity waveforms. For model 3, although an increase in the inlet peak flow is also observed, it is relatively small (6.6%).

### Time-Averaged Wall Shear Stress

Normalized TAWSS distributions using the patient-specific BCs are presented in Figure 9. Areas of high TAWSS_n_ were found at the entrance of the daughter branches. More extended high TAWSS_n_ areas were observed on the LPA and RPA branches of model 3 (Figure 9C), and the RPA and MPA branches of models 4 (Figure 9D) and 7 (Figure 9G). The lowest TAWSS_n_ was noticed in patient 1 (Figure 9A), while the MPA of model 3 had also lower TAWSS_n_ values compared to the rest of the models (Figure 9C). A small area of high TAWSS_n_ was also seen in the MPA of model 5 (Figure 9E).

Overall, the TAWSS_n_ patterns observed in Figures 9 and 6 are similar in character, in particular with regard to the high wall shear stresses developed at the entrance of the daughter branches. Supplementary Table S2 provides the percentage difference between the average and patient-specific flow splits. Smaller areas of high TAWSS_n_ were noticed in models 1–4 (Figures 9A–D), which coincide with increased flow split in the RPA and with an increase in the peak inlet flow (Supplementary Table S2). On the contrary, higher wall shear stress values were developed in the pulmonary bifurcation of models 5–7 (Figures 9E–G) for patient-specific BCs. For models 5 and 6 (Figures 9E,F), there was an increase in the LPA flow split and a decrease in the RPA flow split and peak inlet flow, compared to the average values (Supplementary Table S2). Model 7 had the largest difference in the TAWSS_n_ pattern. A possible explanation could be that although the patient-specific flow split was 45.7:54.3% (*Q*_*RPA*_:*Q*_*LPA*_), the average flow split had most of the flow diverted to the RPA branch (65.3:34.7%, *Q*_*RPA*−*av*_:*Q*_*LPA*−*av*_). Furthermore, model 7 was the exception in the general trend observed for the rest of the models, where an increase in the RPA flow split and the peak inlet flow resulted in increased TAWSS_n_ values in the pulmonary junction.

To quantify the differences between the patient-specific models with patient-specific or averaged boundary conditions, and the average geometry, the time averaged wall shear stress from the cross-sections (α) and (γ) (Figure 1), was compared. Eight points located along the wall of the cross-sections and equally distanced, were taken for each geometry, and the average TAWSS from the eight individual values was calculated. The measured TAWSS was then averaged for Models 1–7, with patient-specific boundary conditions, and for the same models with averaged boundary conditions. The results are presented in Table 5, both in Pascal and non-dimensionalized (TAWSS_n_). The TAWSS values of the averaged geometry is reported separately to allow comparisons. The TAWSS values of the average geometry is lower compared to the average values calculated for Models 1–7 when both averaged and patient-specific BCs are used. In addition, the range of the TAWSS values are comparable when average (~4.7–45 Pa) and patient-specific BCs (~3.3–23.7 Pa) are assigned. Table 5 also indicates that there is a large variability in the TAWSS values between the different models. To further compare the TAWSS values of the different models, the average TAWSS value calculated for the RPA and LPA cross-sections of each patient were plotted and are presented in Figure 10, for both patient-specific and average BCs. When we compare the adult patients (Figure 10A3–7) the TAWSS value was found to be decreasing with age, while the lowest TAWSS value of 2.7 Pa was noticed in model 1 (Figure 10A1). In Figure 10B, much higher TAWSS values were calculated for model 2 (~45 Pa, Figure 10B2), while models 5 to 7 have very similar TAWSS values (Figure 10B5–7). The TAWSS value of the average model was at the lower levels (~6.5 Pa, Figure 10B8), but comparable to the values calculated in the adult population with averaged boundary conditions (4.7–29 Pa, Figure 10B3–7). In general, the TAWSS in the pulmonary bifurcation of the models is found highly increased, which could be the results of the TOF disease.

**Table 5:** Averaged TAWSS, derivd from the TAWSS values of eight points located along the perimeter of cross-sections (α) and (γ), are presented both in Pascal (Pa) and non-dimensionalized (TAWSSn).

		**TAWSS (Pa)**	**TAWSS_**n**_**
Cross-section (α)	Average geometry	8.7	9.5
	Models 1–7 average BCs	18.2 ± 13.5	17.3 ± 17.3
	Models 1–7 patient specific BCs	12.9 ± 6.7	18.7 ± 15.5
Cross-section (γ)	Average geometry	4.2	4.5
	Models 1–7 average BCs	17.9 ± 14.8	15.0 ± 11.0
	Models 1–7 patient specific BCs	10.9 ± 7.0	14.6 ± 11.3

## Discussion

This study demonstrates that the anatomy of the pulmonary arteries of repaired tetralogy of Fallot patients varies greatly within the population. A characteristic difference from healthy subjects is that the left pulmonary artery is highly curved (11). The scope of this study was to evaluate the effect of morphological characteristics on the flow development in models of rTOF patients. Seven patient cases were initially analyzed geometrically and information including the curvature, tortuosity, the radius and the angles of the daughter branches were reported. The flow development was investigated through numerical simulations that were performed using patient-specific and averaged boundary conditions. The novelty of this work lies in the analysis of both the morphological and the hemodynamic characteristics of repaired TOF models, and in the assignment of the patient-specific 3D velocity profile of the pulmonary arteries, which is usually neglected in computational studies. The main findings of this work concern the correlation of tortuosity with the secondary flow patterns (section Secondary Flow), and the association of the flow splits and the peak inlet flow with the time averaged wall shear stress distribution (section Time-Averaged Wall Shear Stress).

The morphological analysis of the TOF models in this study suggests that: (1) the LPA has a higher curvature compared to the RPA; (2) the LPA branch has a higher tortuosity; (3) the average radius of the LPA is higher compared to the RPA radius (Table 3); (4) the in-plane-angles suggest a more acute angle of the LPA branch; and (5) the out-of-plane angles indicate a counter-clockwise and clockwise shift of the RPA and LPA branches, respectively, while also suggesting a small bend of the RPA on the 3D-space.

### Comparison With Previous Studies

Capuano and his co-workers (11) studied the blood flow in a healthy pulmonary artery and reported a steeper but planar curvature in the right pulmonary branch compared to the left, while Louvelle et al. (22) who assessed the geometric complexity of TOF patients, reported higher tortuosity in the LPA, in agreement with our results. Regarding the cross-sectional area between the RPA and the LPA branches, this was previously found slightly lower in the RPA for healthy subjects (11), while the opposite was observed in TOF patients, where the difference between the diameters of the two branches was noticeable (22). In the present study, a small difference was seen between the minimum sphere radius of the RPA and LPA branches. Comparing the branching angles in the right and left branches, the LPA was found to bifurcate almost as a continuation of the MPA in healthy subjects (11), while its course was much different, with smaller branching angles, in TOF patients (22), which is in agreement with what was observed in this work.

To characterize the flow, the Reynolds, Womersley, and Dean numbers were reported in this study, which are associated with alterations in secondary flow structure (38, 39) and wall shear stress patterns (40, 41). The RPA was found to have the highest mean Reynolds number, compared to the MPA and LPA branches, which could be explained by the smaller average branch radius. Finally, the average Dean number was found to be more elevated in the RPA branch, than the LPA branch. The Womersley number reported in the pulmonary arteries of healthy volunteers is in the range of approximately 14–21 (42), which is in agreement with the range reported for the TOF population in this study. Recently, Loke et al. (43) studied the pulmonary artery bending in healthy subjects and patients that have undergone arterial switch operation (ASO), and reported a Dean number in the range of 803 ± 280 in the RPA and 566 ± 140 in the LPA in the control group, and 1,902 ± 1,125 in the RPA and 1,067 ± 584 in the LPA of the ASO patients (43). These patients are often diagnosed with post-operative complications including right ventricular afterload and right ventricular hypertrophy, most commonly from left pulmonary arterial stenosis (44), similar to TOF patients. In our study, the average Dean numbers were within the range of the ASO patients. Plotting the average Dean number along the centrelines of the models showed that it reached its peak value approximately at the entrance of the daughter branches (Figure 4D), following the same trend as the curvature plot (Figure 4B). In general, high Dean numbers are associated with complex vortical flows (45), and a Dean number above 36 was found to be the critical value above which secondary motions are formed in a curved pipe (46).

The flow patterns developed in the arteries of the models are comparable with those reported in previous studies for tetralogy of Fallot patients. Flow recirculation was observed first in the LPA of the models early in the cardiac cycle (Figure 7I), while recirculation zones developed in the MPA and/or RPA branches later during the cardiac cycle in agreement with the study of Chern at al. (21). Various different types of secondary flow patterns have been discussed in the literature (11, 21, 38, 39, 47), for different numerical and geometrical parameters, which makes the comparison with the secondary flow patterns reported in this study rather difficult. Nevertheless, in general, more complicated flow patterns were found in the LPA branch (Figure 8) of the models, which is in agreement with other studies (21). Finally, the TAWSS_n_ distribution was, overall, elevated at the openings of the daughter branches (Figures 6, 9), which is comparable with the results reported by Zhang et al. (19). Very low TAWSS_n_ values were found at the stagnation point, while a high TAWSS_n_ gradient was developed adjacent to that area, as reported also by Boumpouli et al. (23, 48). The range of TAWSS values reported in this study for the rTOF models (in the range of 0–44 Pa, Figure 10) was found much higher compared to those of healthy control volunteers presented in previous studies (up to 2.05 Pa), and of patients with pulmonary arterial hypertension (up to 1.01 Pa) (13, 49). Nevertheless, the range of the TAWSS values in Zhang et al. (19), a work also focused in rTOF, was also elevated, at 0–100 Pa (19) and the authors anticipated that the pulmonary arteries of the specific population of patients is characterized by a high TAWSS environment, as a result of the underlying disease condition. In general, higher wall shear stress values were observed in the RPA branch, which was also characterized with a smaller branch radius and higher *Re* and *De* numbers.

### Importance of Patient-Specificity

Anatomically mean geometries have been used in several cardiovascular studies to reduce complexity in patient-specific simulations (50), investigate morphological characteristics and extract 3D shape biomarkers (31), and, lately, study the blood flow in a healthy average geometry of the pulmonary bifurcation derived from 5 young patients (11). To this end, we compared our patient-specific results with those of an averaged geometry derived from our 5 adult rTOF patients. The regions of high TAWSS_n_ (Figure 6H) were in agreement with those predicted with the patient-specific models (Figures 9A–G), more closely resembling models 1, 2, and 6 (Figures 9A,B,F). Nevertheless, the extent of the high shear regions differed considerably with morphology and an anatomically mean geometry cannot capture this variability. This study also demonstrates a similarity in character between results obtained with patient specific boundary conditions and those utilizing averaged conditions. This indicates that morphology is a crucial parameter in the flow development. However, the TAWSS_n_ distribution varied in cases with the highest percentage difference in flow split and peak inlet flow between patient-specific and averaged values (Supplementary Table S2), particularly for model 7 (Figure 6G) where this was more visible. In models 5 and 6, where the flow split in the RPA branch and peak inlet flow increased with the patient-specific boundary conditions, higher values of TAWSS_n_ were observed (Figures 9E,F). A correlation therefore exists between the TAWSS_n_ distribution and flow splits which has been previously reported in idealized models, representative of the pulmonary bifurcation (51). Finally, the results of Figure 10 indicate a decrease in the TAWSS in the cross-sections of the RPA and LPA branches with age, with the exception of patient 1, the youngest patient in our group when the patient-specific BCs are considered (Figure 10A).

Patient-specificity is therefore deemed necessary for a better characterization of the flow development in the pulmonary bifurcation of rTOF patients. Geometric analysis of the patient-specific models provides information about curvature and tortuosity, parameters that affect the flow in the daughter branches, and more specifically recirculation (Figure 7) and secondary flows (Figure 8). Curvature and torsion that exist in arterial geometries are found to create a favorable environment through generation of mixing, which has been linked with thrombosis prevention (52). On the other hand, Dean flow patterns are referred to as no mixing, as the two vortices formed are symmetric (52). In addition, assuming patient-specific boundary conditions is also important, as the flow split and peak inlet flow have an impact on the TAWSS_n_ distribution (Figures 6, 9), and on the secondary flow vortices developed on localized cross-sections (Figures 5, 8). Significant changes on the wall shear stress of the pulmonary vasculature could affect pulmonary remodeling and endothelial health (49). Although similarities existed between the TAWSS_n_ of the patient-specific and averaged boundary conditions, and one would argue that it could suffice to predict the wall shear stress patterns, model 7, in particular, highlights the value of patient-specificity.

### Clinical Relevance

The reversal of blood flow into the right ventricle, known as pulmonary regurgitation, and branch pulmonary artery stenosis, in the form of LPA kinking, are two of the most common complications in the rTOF population. PR is the result of pressure difference between the right ventricle and the pulmonary artery during diastole, and is associated with the geometry of the branch pulmonary arteries, and with the pulmonary vascular resistance and pressure (53, 54). Chronic PR typically results in right ventricular dilatation and dysfunction, and in combination with the dilation of the pulmonary trunk, the RVOT is restricted and turns cranially and left-laterally resulting in an acute angle between the MPA and the LPA (53, 55). Branch stenosis is associated with increased PR, increased pressure drop, restricted flow to the corresponding lung and with increased retrograde flow in the larger artery leading to increased pulmonary vascular resistance in the non-stenotic branch (54, 56–58).

Endothelial cells (ECs) and elastic fibers are organized in a layer of connective tissue that surrounds the endothelial lining of the tunica intimate (inner layer) of the blood vessels (59, 60). ECs play a crucial role in regulating vascular homeostasis (61, 62) and remodel in response to the wall shear stress applied to the vessel's wall. Increased levels of WSS increase the production of the potent vasodilator nitric oxide (NO), while decreased WSS increase the production of the potent vasoconstrictor endothelin-1 (ET-1) (63–65). In addition, TAWSS values <0.4 Pa are linked to atherosclerosis, while higher TAWSS (15–45 Pa) with thrombosis (66). Pulmonary arteries have high shear stress compared to other arteries, but changes observed in the pulmonary vasculature could still affect endothelial function and remodeling (49). The results of this study indicate that the TAWSS in the pulmonary arteries of the rTOF patients is highly increased, with values up to 24 Pa (when the patient-specific boundary conditions are considered) at the entrance of the daughter branches and with slightly higher TAWSS values noticed at the RPA branch. These regions of sudden change in the TAWSS at the bifurcation of the pulmonary artery may be potential areas of local stenosis and neointimal growth (19). The hemodynamic environment of these patients could be potentially correlated with the two-way link between pulmonary regurgitation and LPA kinking, were when present, may lead to RV dilation and dysfunction.

### Limitations

Several limitations exist in the specific study, including the assumption of rigid walls. Previous studies have indicated that the flow patterns observed with the rigid walls would not differ significantly from those predicted when dynamic arterial compliance is taken into account (11, 67). A maximum wall displacement of 0.064 cm was reported in the pulmonary bifurcation of a patient-specific Fontan configuration model (68), which is very close to the 0.054 cm of maximum displacement reported by the authors in another study (69), accounting for ~2.6% change in the diameter of the vessel and 5.8% difference at the integral of velocity based on simulations with fluid-structure interaction methods (23, 69). In addition, the peripheral resistance of the vessels was neglected, although patient-specific flow splits were assigned at the outlets of the models. Also, the pulmonary valve was not included in the simulations, which would affect the inlet flow, however patient-specific 3D velocity profiles were assigned at the inlet of the models which are more representative compared to a pulsatile waveform. Furthermore, the authors recognize that the anatomical mean geometry cannot be considered representative of the rTOF patients, due to the small number of models used in the study. Finally, specific disease indications of the rTOF patients are not available, which consist another limitation of this study. Future work will include a larger cohort of patients to verify the findings presented and to address some of the limitations. Finally, it is acknowledged that the examined cohort of patients varied greatly in terms of age and gender, which may influence particularly the averaged BCs and geometry used.

## Conclusion

This study investigated the impact of morphological characteristics in the blood flow development in the pulmonary arteries of rTOF patients, assuming patient-specific and averaged boundary conditions. Higher curvature and tortuosity were found on the LPA branch, which also formed a more acute angle with the MPA and had a more pronounced rotation in the 3D space. Zones of recirculation and more complex flow patterns also developed in the specific daughter branch as indicated by the computational results. The LPA was characterized by lower Reynolds and Dean numbers, and the results correlated with higher curvature and tortuosity of the branch. Nevertheless, the higher Re and De numbers, which imply more distributed flow in the domain, can correlate with increased regions of TAWSS_n_ in the RPA branch. The averaged boundary conditions and the average geometry can capture some general characteristics of the flow, supporting the importance of morphology in flow development. The present work highlights the importance of patient-specificity and especially of the spatial varying flow, which is usually neglected in computational studies. Further investigation is required in larger cohorts of TOF patients to validate the findings of this study and to allow the analysis based on the age and gender of the population.

## Data Availability Statement

The raw data supporting the conclusions of this article will be made available by the authors, without undue reservation.

## Ethics Statement

The studies involving human participants were reviewed and approved by the use of retrospectively collected image data for research purposes was approved by the Institute of Child Health/Great Ormond Street Hospital Research Ethics Committee, and written consent was obtained from all subjects or parents/legal guardians (Ref: 06/Q0508/124). Written informed consent to participate in this study was provided by the participants' legal guardian/next of kin.

## Author Contributions

MB: formal analysis, methodology, software, writing—original draft, and visualization. AK, CC, ES, and MB: conceptualization, investigation, data curation, writing—review and editing, and project administration. AK, CC, ES, and SS: resources, writing—review and editing, supervision, and funding acquisition. All roles according to CRediT (contributor roles taxonomy). All authors contributed to the article and approved the submitted version.

## Funding

This work was supported in part from the UK Research and Innovation (UKRI) Engineering and Physical Sciences Research Council (EPSRC), under grant agreement EP/N032861/1 through a Short Research Visit (SRV) funding, the University of Strathclyde Research Studentship Scheme (SRSS) Research Excellence Awards (REA), Project No. 1208 and the EPSRC grant EP/N02124X/1. ES was supported by a Research Training Fellowship (GN2572) granted by Action Medical Research. CC was supported by the British Heart Foundation (PG/17/6/32797). SS was supported by an ERC Starting Grant (ERC-2017-StG-757923). AK was supported by the European Union's Horizon 2020 research and innovation programme under the Marie Skłodowska-Curie grant agreement No. 749185.

## Conflict of Interest

The authors declare that the research was conducted in the absence of any commercial or financial relationships that could be construed as a potential conflict of interest.

## Publisher's Note

All claims expressed in this article are solely those of the authors and do not necessarily represent those of their affiliated organizations, or those of the publisher, the editors and the reviewers. Any product that may be evaluated in this article, or claim that may be made by its manufacturer, is not guaranteed or endorsed by the publisher.

## Nomenclature

ASO: Arterial switch operation
BCs: Boundary conditions
CT: computed tomography
CSV: Comma-separated value
De: Dean number
LPA: Left pulmonary artery
MPA: Main pulmonary artery
MR: Magnetic resonance
MRI: Magnetic resonance imaging
PR: Pulmonary regurgitation
rTOF: repaired Tetralogy of Fallot
Re: Reynolds number
RPA: Right pulmonary artery
RVOT: Right ventricular outflow tract
STL: stereolithography
TAP: Transannular patch repair
TAWSS: Time averaged wall shear stress
Wo: Womersley number.
