# Supplementary material for: Characterization of Flow Dynamics in the Pulmonary Bifurcation of Patients With Repaired Tetralogy of Fallot: A Computational Approach
Source: Front Cardiovasc Med. 2021 Sep 30;8:703717. doi: 10.3389/fcvm.2021.703717 (PMC8514754; doi:10.3389/fcvm.2021.703717)
Supplement: Supplementary file 1 [file Data_Sheet_1.pdf]

Supplementary Material

A more detailed description of the results of this study is provided in the following sections. Section 1 is an extension of the geometry characterization, while section 2 of the flow characterization. The values used for the non-dimensionalization of the TAWSS and the percentage of difference between the average and patient-specific values for the flow splits and the inlet flow, are also reported.

1 Geometry Characterization

To further extend the geometric analysis presented in Section Geometry Characterization in Results, the minimum, mean and maximum values of torsion were calculated and are reported on the supplementary Table S1, for all seven patients. The results indicate higher torsion in the RPA branch of the models, with the maximum average value of 0.135 mm^{-1} observed in the RPA of patient 1. The mean torsion was found very close to zero for both daughter branches.

Table S1: Torsion analysis of the patient-specific models

Patient	Torsion RPA (mm^{-1}) (min / mean / max)	Torsion LPA (mm^{-1}) (min / mean / max)_
1	-0.430 / 0.135 / 1.798	-0.074/ 0.024 / 0.182
2	-0.500 / -0.055 / 0.183	-0.864 / -0.138 / 0.005
3	-0.400 / -0.004 / 0.841	-0.971 / 0.026 / 0.742
4	-1.017 / -0.030 / 0.367	-0.046 / 0.059 / 0.837
5	-1.338 / -0.032 / 0.709	-2.083 / -0.061 / 0.606
6	-0.008 / 0.128 / 0.495	-0.888 / -0.004 / 0.294
7	-0.329 / 0.004 / 0.709	-0.343 / 0.009 / 0.930
Mean value	-0.575 / 0.021 / 0.729	-0.753 / -0.012 / 0.514

2 Flow Characterization

To complement the analysis presented at the paper, additional secondary flow patterns for the remaining of the models, with patient-specific boundary conditions, and the oscillatory shear index (OSI) distribution, are included. OSI is an indicator of the alteration of the wall shear stress (WSS) at the vessel's wall, and together with WSS have been correlated with endothelial function in the pulmonary arteries (1).

The difference between the average and patient-specific values for the flow splits and inlet flow on supplementary Table S2 and the TAWSS value at the inlet wall for each model is given on supplementary Table S3.

Table S2: Percentage difference between the average and patient-specific values for flow splits and inlet flow waveform ((($Q_{average}$-$Q_{patient\_specific}$)/$Q_{average}$)*100).

Patient	Flow Split % difference (RPA)	Flow Split % difference (LPA)	Inlet flow Waveform % difference	Peak Inlet flow % difference
1	+15.3%	-28.8%	+55.5%	+51.6%
2	+0.8%	-1.4%	-3.2%	+54.5%
3	+15.0%	-28.0%	+57.7%	+6.6%
4	+2.1%	-4.0%	-10.8%	+6.5%
5	-15.5%	+29.1%	-166.3%	-94.8%
6	-17.3%	+32.6%	+68.4%	-52.6%
7	+30.0%	-56.5%	-1.2%	+9.0%

Table S3: TAWSS value at the inlet of the models when average and patient-specific boundary conditions (BCs) are assigned.

Patient	TAWSS (Pa)	
	Average BCs	Patient-specific BCs
1	4.2	2.0
2	2.0	1.6
3	1.0	0.6
4	1.4	1.3
5	1.2	1.0
6	0.2	0.7
7	1.6	0.2
Average geometry	0.9	-

2.1 Secondary flow

2.1.1 Average boundary conditions

A more detailed analysis of the secondary flow in cross sections (α) and (γ) along the RPA and LPA of Fig. 5 of the paper is provided. The description of the flow patterns is based on Perry and Steiner's (2). Looking at the orientation of the streamlines, the terms stable and unstable refer to the vectors of flow pointing towards the inner and outer of a node, focus or line, respectively, while a saddle point is a location where four streamlines form a rhombus of a stationary region.

During peak flow, a stable node was noticed in the RPA of all models (Fig. 5AI (α), 5BI (α), 5CI (α)). In addition, two pairs of counter rotating vortices were observed in the LPA of model 2 (Fig. 5AI (γ)). The pair positioned cranial and posteriorly, consisted of a stable and an unstable focus, while the other pair was positioned caudally in the LPA slice, and consisted of two unstable foci. In model 3, an unstable bifurcation line was visible in the LPA (Fig. 5BI (γ)), while for the average model a stable

bifurcation line positioned cranially, and an unstable focus positioned caudally were developed (Fig. 5CI (γ)).

During mid deceleration at systole, the stable bifurcation line in the RPA of model 2 developed in a stable focus (Fig. 5AII (α)) while in the average model two counter rotating vortices are additionally developed posteriorly (5CII (α)). In the LPA of model 2, only three vortices remained; a stable focus positioned cranially, and two unstable foci positioned caudal anteriorly and posteriorly, respectively (Fig. 5AII (γ)). A saddle was visible cranially and anteriorly in the LPA of model 3, with an unstable focus further caudally and posteriorly and a stable focus caudally (Fig. 5BII (γ)). For the average model, 2 vortices, positioned cranially and caudally were developed, while a saddle was also visible cranially, during mid diastole (Fig. 5CII (γ)).

At mid diastole, two unstable foci appeared cranially in the RPA of model 2, and one located more centrally (Fig. 5AIII (α)). For the LPA, an unstable bifurcating line, and an unstable focus were visible cranially and caudally, respectively (Fig. 5AIII (γ)). In model 3, an unstable bifurcating line was visible in the RPA cross-section (Fig. 5BIII (α)), while a pair of counter-rotating vortices, consisting of two stable foci, positioned caudally in the LPA cross-section (Fig. 5BIII (γ)). For the average model, an unstable bifurcating line appeared posteriorly in the RPA (Fig. 5CIII (α)), while in the LPA a stable bifurcating line and an unstable focus were formed caudally and cranially, respectively (Fig. 5CIII (γ)).

2.1.2 Patient-specific boundary conditions

Starting with the description of the vortices for models 2 and 3, presented in Fig. 8 of the paper, two counter rotating vortices, were visible in the LPA of model 2 (Fig. 8AI (γ)), which then disappeared further downstream, and an unstable bifurcation line was instead visible (Fig. 8AI (δ)), during peak flow. The vortices were not symmetric, and the one positioned posteriorly was enlarged (unstable focus). The asymmetry of the vortices is expected due to the higher curvature and the velocity profile of the flow entering the bifurcation. In model 3, a stable focus was formed posteriorly (Fig. 8BI (γ)), which remained further downstream (Fig. 8BI (δ)), while a saddle point was also developed cranially (Fig. 8BI (δ)). In the RPA branches, a bifurcation line was initially observed in both models 2 and 3 (Fig. 8AI (α), 8BI (α)), which developed further downstream into an unstable focus (Fig. 8AI (β)) with reversed flow (Fig. 8AI (β), 8BI (β)).

During mid-deceleration, two unstable foci located posteriorly, a stable focus and a saddle point located anteriorly, were formed in the LPA cross-section of model 2 (Fig. 8AII (γ)), which further downstream fused into one stable focus moving cranially (Fig. 8AII (δ)). In the LPA of model 3, two relatively symmetric vortices were visible (Fig. 8BII (γ)), which moved cranially downstream (Fig. 8BII (δ)), with an additional vortex formed in the caudal position. In the RPA both models 2 and 3 a stable focus was visible, located centrally in model 2 (Fig. 8AII (α)), which then moved posteriorly (Fig. 8AII (β)), and posteriorly in model 3 (Fig. 8BII (α)), subsequently moving cranially (Fig. 8BII (β)). A stable bifurcation line also existed cranially in the RPA of model 3 (Fig. 8BII (α)).

A pair of counter-rotating vortices appeared during mid diastole in the RPA of model 3, positioned caudally in the upstream cross-section (Fig. 8BIII (α)), but then moving posteriorly further downstream (Fig. 8BIII (β)). A stable focus existed in the RPA of model 2 at both cross-section (Fig. 8AIII (α, β)). In the LPA branch, a stable and an unstable focus were visible in the cross-section of model 2 (Fig. 8AIII (γ)), which fused into a stable focus downstream (Fig. 8AIII (δ)). The opposite was observed for model 3, where a stable focus (Fig. 8BIII (γ)), transitioned to two counter-rotating vortices in the downstream cross-section (Fig. 8BIII (δ)).

Contours of velocity normal to the cross sections of the rest of the models (1, 4, 5, 6 and 7) are presented in the supplementary Fig. S1-3, overlaid with the in-plane velocity vectors at peak systole, mid deceleration of systole and mid diastole.

During peak flow, no vortices were present in the RPA branch of any of the models (Fig. S1). A stable bifurcation line was formed, which developed into a saddle point on model 7 (Fig. S1E (β)). In the LPA branches of the models, a pair of counter-rotating vortices was visible in model 4 and 6, located posteriorly and caudally, respectively. Both pairs consisted of a stable and an unstable focus (Figs. S1B (γ), S1D (γ)). The vortices in model 4, moved anteriorly further downstream (Fig. S1B (δ)). A small vortex was visible cranially in model 1 (Fig. S1A (γ)), and two vortices, cranially and anteriorly, and a bifurcation line were noticed in model 5 (Fig. S1C (γ)). For the remaining of the models, stable bifurcation lines were observed (Figs. S1 (γ)-(δ)).

During mid deceleration at systole, vortices were apparent in the majority of the models, in both the RPA and LPA branches. A stable bifurcation line in model 1 (Fig. S2A (α)), developed into a stable focus (Fig. S2A (β)), while in model 4 the stable focus was formed in (α) and moved posteriorly in (β) (Fig. S2B), as is in model 5 (Fig. S2C). In model 7 a saddle point and a stable focus were observed (Fig. S2E). Models 6 was the only model with two counter rotating vortices formed on the RPA branch (Fig. S2D (β)). More complex flow patterns were visible in the LPA branches of the models. An unstable focus positioned caudally and anteriorly in model 1 (Fig. S2A (γ)) developed in two counter rotating vortices (Fig. S2A (δ)). Model 4 had two unstable foci caudally, and another vortex consisting of a stable focus cranially (Fig. S2B (γ)), which they remained in (δ). Model 5 had a single vortex and a stable bifurcation line (Fig. S2C (γ)), while model 6 had two counter-rotating vortices caudally (Fig. S2D (γ)) that were fused into one further downstream; a stable bifurcating line also was visible (Fig. S2D (δ)). Model 7 had two relatively small counter-rotating vortices cranially and a stable bifurcation line caudally (Fig. S2E (γ)).

In mid diastole, unstable bifurcating lines were observed in the RPA branch in the majority of models. Vortices consisting of stable foci were visible in model 5 (Fig. S3C (β)), model 6 (Fid. S3D (β)) and model 7 (Fig. S3E (β)). In model 6 three vortices and a saddle point were initially formed (Fig. S3D (α)), while in model 7 five vortices were seen (Fig. S3E (α)). For the LPA branch of the models, vortices were visible in most of the models (Figs. S3A (γ)-(δ), S3B (γ), S3C (γ), S3E). Additionally, unstable bifurcating lines could be distinguished (Figs. S3A (γ), S3B (γ), S3C (γ)).

The results suggest that more complex flow patterns are developed in the LPA branch, already stated in the paper, and is therefore correlated with the higher curvature and tortuosity of the left pulmonary branch. Nevertheless, a number of vortices have formed in the RPA branch during mid diastole, especially in models 6 and 7 (Figs. S3D, S3E), which are also the models with the highest RPA flow split (above 75% in both cases).

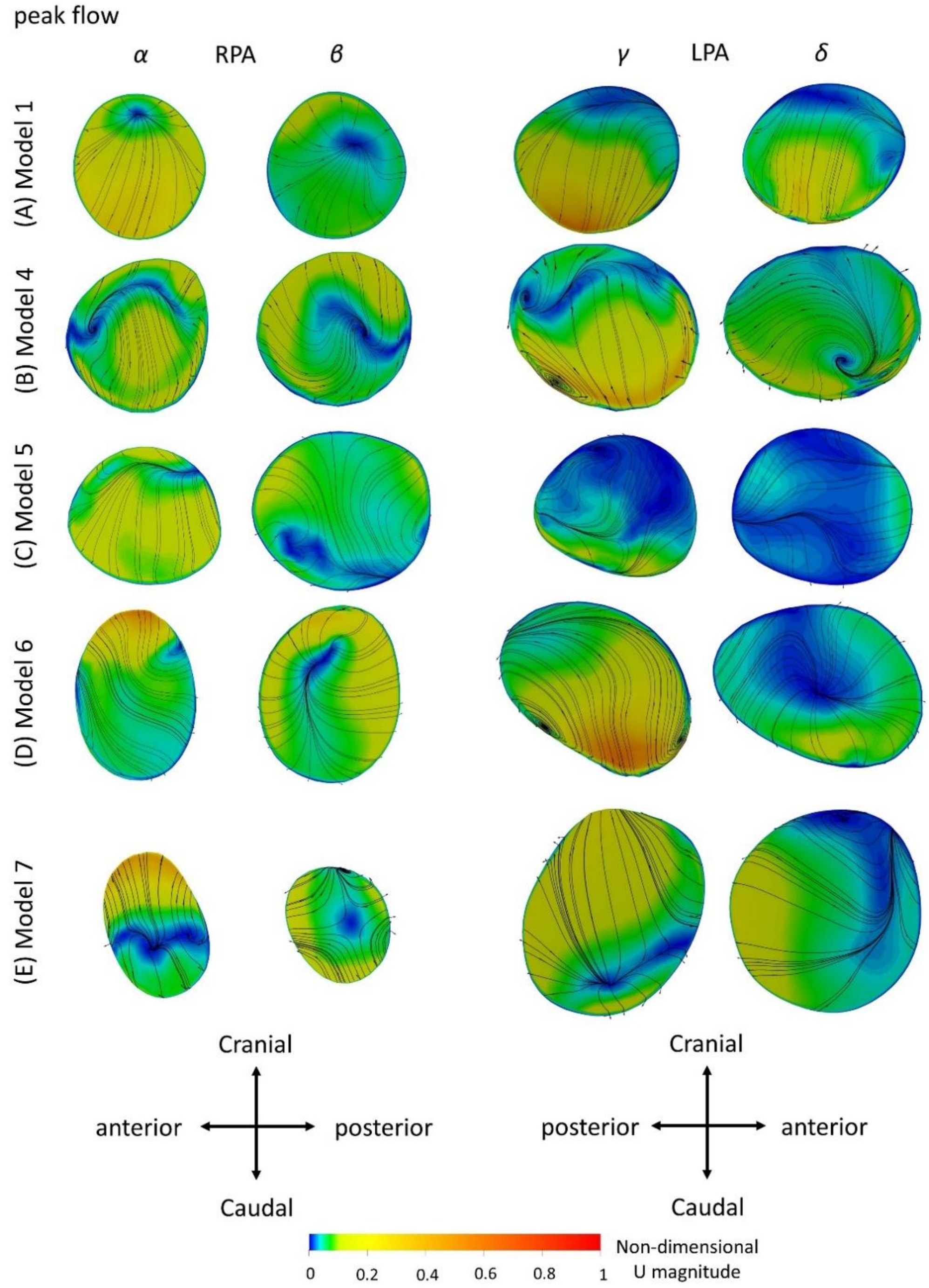

Figure S1. Secondary flow visualised by in plane velocity vectors and contours of normalized velocity normal to the slice during peak flow, for (A) Model 1; (B) Model 4; (C) Model 5; (D) Model 6; and (E) Model 7. Non-dimensionalization was performed by division with the maximum velocity of each patient during the cardiac cycle. Points where slices (α) to (δ) are taken are visible in Fig. 1 of the paper. Cross-sections are oriented with the top and the bottom edges corresponding to the cranial and caudal positions, respectively and left and right to the anterior and posterior of the pulmonary artery, for the RPA, and to the posterior and anterior of the pulmonary artery, for the LPA, respectively. Cross-sections are in scale.

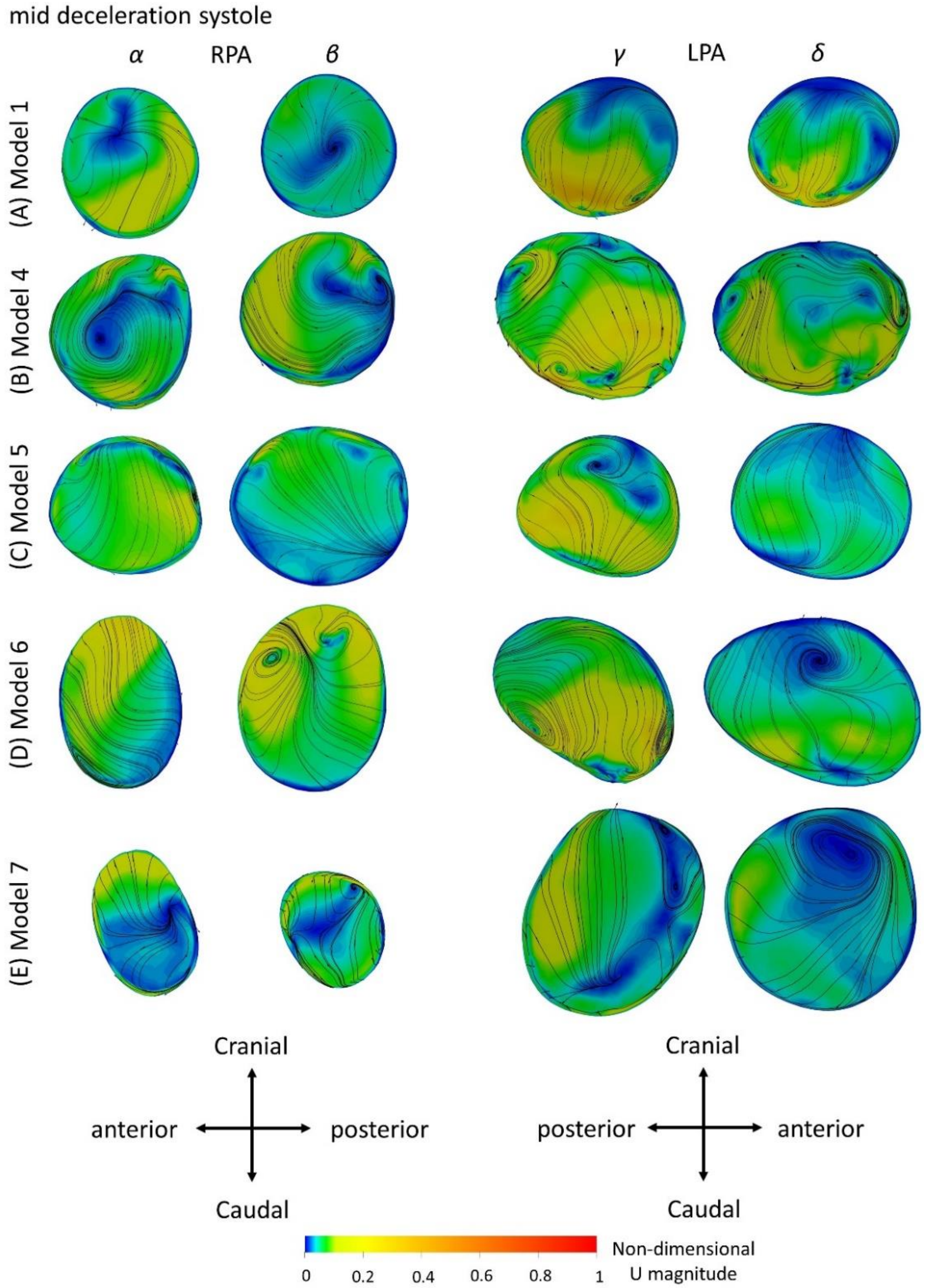

Figure S2. Secondary flow visualized by in plane velocity vectors and contours of normalized velocity normal to the slice during mid deceleration at systole, for (A) Model 1; (B) Model 4; (C) Model 5; (D) Model 6; and (E) Model 7. Non-dimensionalization was performed by division with the maximum velocity of each patient during the cardiac cycle. Points where slices (α) to (δ) are taken are visible in Fig. 1 of the paper. Cross-sections are oriented with the top and the bottom edges corresponding to the cranial and caudal positions, respectively and left and right to the anterior and posterior of the pulmonary artery, for the RPA, and to the posterior and anterior of the pulmonary artery, for the LPA, respectively. Cross-sections are in scale.

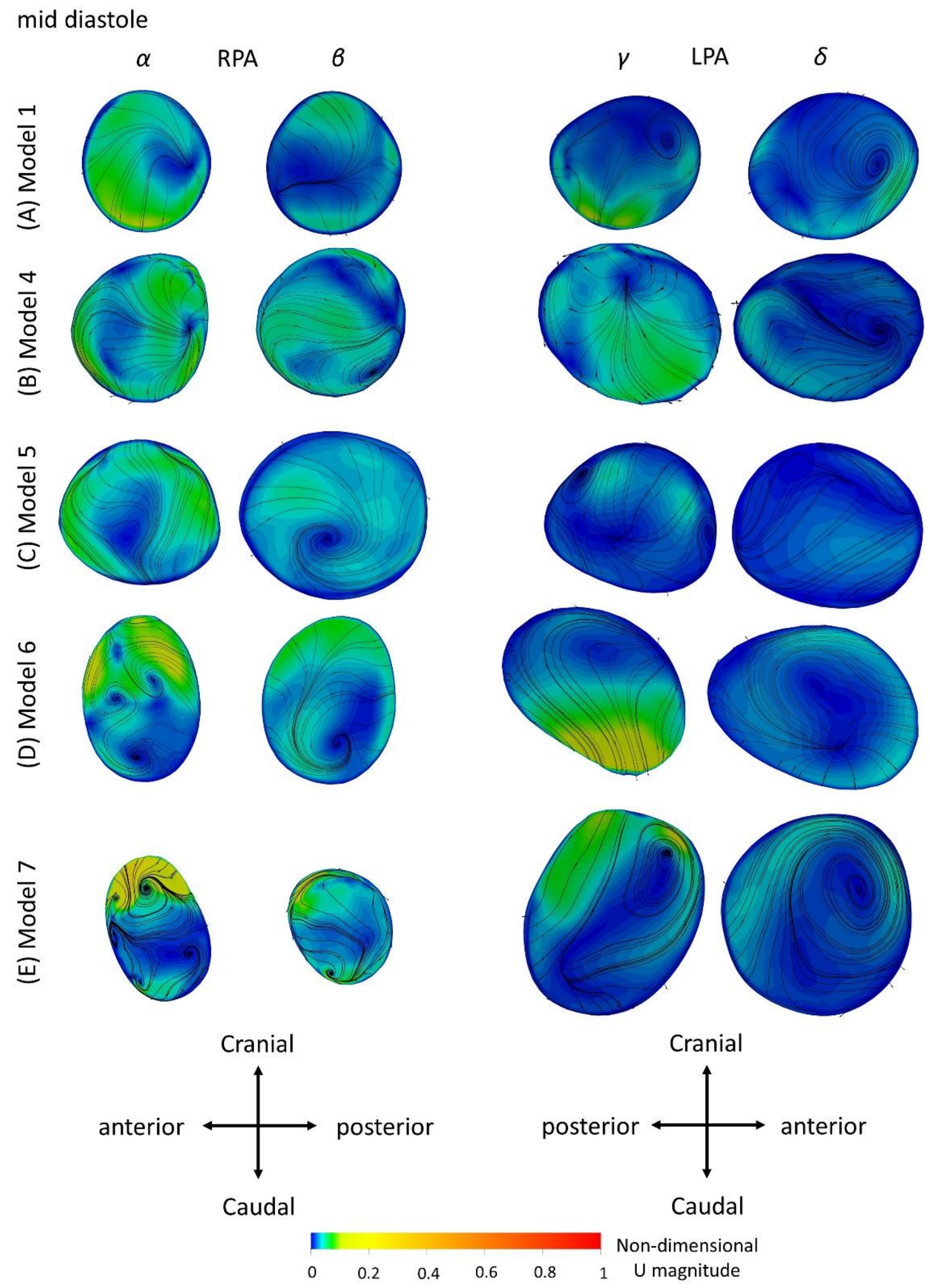

Figure S3. Secondary flow visualised by in plane velocity vectors and contours of normalized velocity normal to the slice during mid diastole, for (A) Model 1; (B) Model 4; (C) Model 5; (D) Model 6; and (E) Model 7. Non-dimensionalization was performed by division with the maximum velocity of each patient during the cardiac cycle. Points where slices (α) to (δ) are taken are visible in Fig. 1 of the paper. Cross-sections are oriented with the top and the bottom edges corresponding to the cranial and caudal positions, respectively and left and right to the anterior and posterior of the pulmonary artery, for the RPA, and to the posterior and anterior of the pulmonary artery, for the LPA, respectively. Cross-sections are in scale.

2.2 Oscillatory Shear Index

In addition to the time averaged wall shear stress, the oscillatory shear index (OSI) was also calculated and is presented in the supplementary Fig. S4, for all the models, using the following formula:

$$OSI = \frac{1}{2}\left(1 - \frac{\left|\frac{1}{T}\int_0^T t_S dt\right|}{\frac{1}{T}\int_0^T |t_S| dt}\right)$$

where T the period of the cardiac cycle and t_s the tangential component of the traction vector. The OSI represents the level of disturbed flow and takes values from 0 (indicating unidirectional shear stress through the cardiac cycle) to 0.5 (corresponds to an average shear stress value of zero during the cardiac cycle) (3).

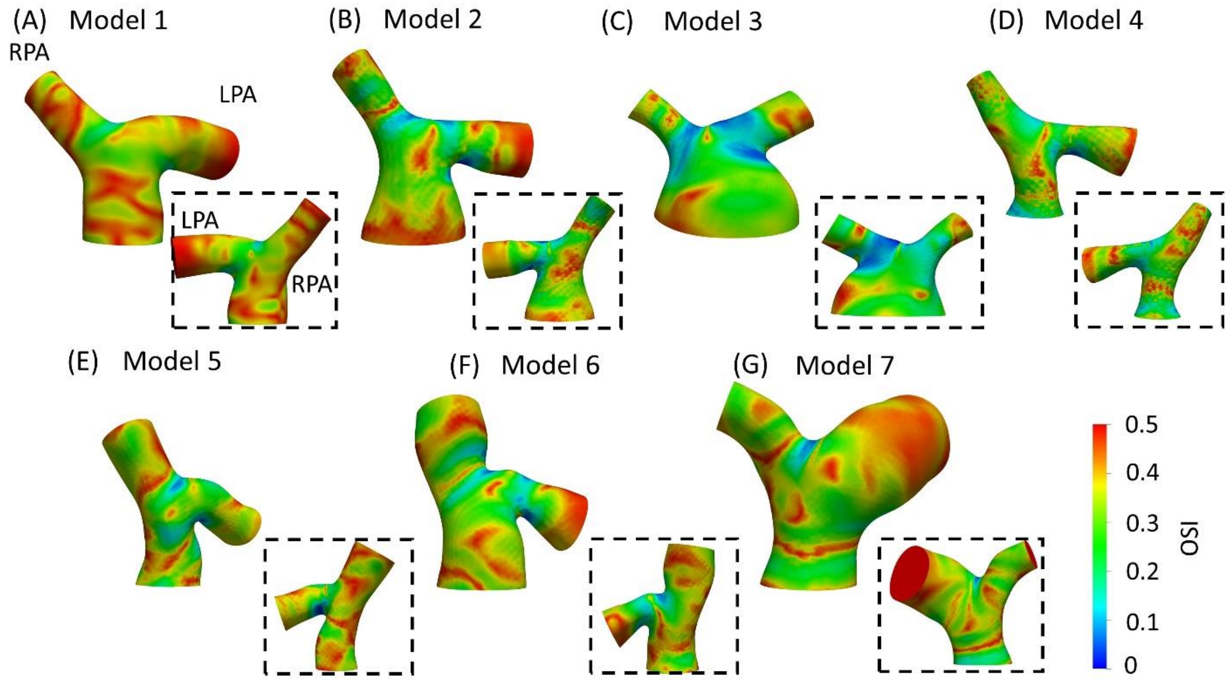

Figure S4. Oscillatory Shear Index distribution for A. Model 1, B. Model 2, C. Model 3, D. Model 4, E. Model 5, F. Model 6 and G. Model 7. The RPA and LPA branches are indicated in model 1.

Relatively high OSI was observed in the pulmonary junction of the models, indicating a fluctuation in the wall shear stress during the cardiac cycle. Lower OSI was seen at the entrance of the daughter branches, and a more extended low region was found in model 3 (Fig. S4C). In general, the high and low OSI regions corresponded to the low and high $TAWSS_n$ areas, respectively (as presented in Fig. 9 of the paper). Previous work conducted in healthy subjects, reported low oscillatory shear index in the pulmonary arterial models (3), while in another study where they compared the OSI in a healthy population with a group of patients suspected of secondary pulmonary arterial hypertension (PAH), higher OSI values were reported for the PAH group (1). The latter is in agreement with the results of this study.

References

1. Terada M., Takehara Y., Isoda H., Uto T., Matsunaga M., Alley M. Low WSS and High OSI Measured by 3D Cine PC MRI Reflect High Pulmonary Artery Pressures in Suspected Secondary Pulmonary Arterial Hypertension. Magn Reson Med Sci. (2016) 15:193-202.
2. Perry AE, Steiner TR. Large-scale vortex structures in turbulent wakes behind bluff bodies. Part 1. Vortex formation processes. Journal of Fluid Mechanics. (2006) 174:233-270.
3. Tang B.T., Fonte T.A, Chan F.P., Tsao P.S., Feinstein J.A., Taylor C.A. Three-Dimensional Hemodynamics in the Human Pulmonary Arteries Under Resting and Exercise Conditions. Annals of Biomedical Engineering. (2011) 39:347-358.
